# Meta-Pyridyl Substitution for New Blue Host Materials with Enhanced Thermal Stability and Electron-Injection Properties

**DOI:** 10.3390/molecules31203768

**Published:** 2026-10-20

**Authors:** Jeongmin Han, Sangwook Park, Saeyoung Oh, Hyeonho Park, Kiho Lee, Hayoon Lee, Jongwook Park

**Affiliations:** Integrated Engineering, Department of Chemical Engineering, Kyung Hee University, Yongin-si 17104, Gyeonggi-do, Republic of Korea; jmhan123@khu.ac.kr (J.H.); pswook@khu.ac.kr (S.P.); tpdud5821@khu.ac.kr (S.O.); dondiamore@khu.ac.kr (H.P.); kiholee@khu.ac.kr (K.L.); kssarang1@khu.ac.kr (H.L.)

**Keywords:** OLED, blue, host, multiple-resonance, pyridine

## Abstract

Two new host materials, 3-(3-(2,12-di-tert-butyl-5,9-dioxa-13b-boranaphtho[3,2,1-de]anthracen-7-yl)phenyl)pyridine (TDBA-Py) and 3,3′-(5-(2,12-di-tert-butyl-5,9-dioxa-13b-boranaphtho[3,2,1-de]anthracen-7-yl)-1,3-phenylene)dipyridine (TDBA-dPy), were designed and synthesized by introducing one pyridine unit at the meta position and two pyridine units at the 3,5-positions, respectively, of the terminal phenyl ring of the TDBA core, a 5,9-dioxa-13b-boranaphtho[3,2,1-de]anthracene (DOBNA)-based multiple-resonance framework, and the resulting changes in their properties were analyzed. By controlling the number of pyridine substituents at the meta positions of a single phenyl ring, the extension of intramolecular conjugation was suppressed, which maintained a high triplet energy (T_1_ ≥ 2.76 eV) and the emission wavelength and simultaneously improved the electron-injection properties. In the devices employing ν-DABNA as the dopant, the TDBA-Py-based device exhibited a low turn-on voltage of 2.5 V, a maximum power efficiency (PE_max_) of 10.9 lm W^−1^, and a maximum current efficiency (CE_max_) of 12.5 cd A^−1^, which can be attributed to its lowered lowest unoccupied molecular orbital (LUMO) level. These results demonstrate that introducing appropriate electron-accepting character through single pyridine substitution is an effective host-design strategy for improving device efficiency.

## 1. Introduction

High-performance organic light-emitting diodes (OLEDs) require balanced hole and electron transport within the emitting layer (EML), which enhances the efficiency of exciton formation through charge recombination. In this context, multicomponent emissive systems that combine a hole-transporting p-type host with an electron-transporting n-type host have been adopted in academic and industrial research as an alternative to single-host systems [1,2]. The p-type host facilitates hole injection and transport, whereas the n-type host facilitates electron injection and transport. Exciplex formation between these two hosts can reduce charge-injection barriers and stabilize excited states, thereby improving charge balance in the emissive layer and potentially extending device lifetime.

P-type hosts commonly incorporate moieties such as carbazole, diphenylamine, and triphenylamine to achieve high triplet energies and efficient hole transport. P-type hosts incorporating these moieties have been widely studied and commercialized. Representative carbazole-based p-type hosts include 1,3-bis(N-carbazolyl)benzene (mCP), 4,4′-bis(N-carbazolyl)-1,1′-biphenyl (CBP), and 3,3′-di(9H-carbazol-9-yl)-1,1′-biphenyl (mCBP). Examples based on diphenylamine and triphenylamine derivatives include 1,1-bis[(di-4-tolylamino)phenyl]cyclohexane (TAPC) and tris(4-carbazol-9-ylphenyl)amine (TCTA) or 4,4′,4″-tris[(3-methylphenyl)phenylamino]triphenylamine (m-MTDATA), respectively [3,4,5]. Common electron-withdrawing units for n-type hosts include heteroaromatic moieties such as triazine and pyridine, as well as functional groups such as cyano and phosphine oxide groups. Typical n-type hosts based on these units include the triazine-based 2,4,6-tris(biphenyl-3-yl)-1,3,5-triazine (T2T), the pyridine-based 1,3,5-tri(m-pyridin-3-ylphenyl)benzene (TmPyPB), the cyano-containing 3,3′-di(carbazol-9-yl)-5-cyano-1,1′-biphenyl (mCBP-CN), and the phosphine oxide-based bis[2-(diphenylphosphino)phenyl] ether oxide (DPEPO) [4,6,7,8,9].

However, the development of high-performance n-type hosts that combine the high energy levels required for blue emission with efficient electron injection and transport and robust thermal stability remains a significant challenge. Compared with p-type hosts, n-type hosts have been more difficult to develop for several reasons. The introduction of electron-withdrawing moieties tends to lower the energy of the lowest excited triplet state (T_1_) level, and the resulting materials often suffer from intrinsic instability. When electron-withdrawing moieties are introduced to facilitate electron transport, conjugation is often extended through phenylene bridges or similar linkers, which leads to a decrease in the triplet energy [10]. Suppressing the extension of π-conjugation to prevent this reduction in triplet energy can also impair electron transport. Thus, n-type host design is generally considered to involve a trade-off between T_1_ and charge-transport capability [11,12]. Phosphine oxide-based hosts, which are commonly used to achieve high T_1_ levels, have been reported to exhibit limited chemical stability owing to the relatively unstable C–P bond. This instability can lead to degradation during thin-film formation and device operation [13].

To address both the trade-off between T_1_ and electron transport and the chemical instability, a multiple-resonance (MR) framework was selected as the backbone in this study. Unlike conventional donor–acceptor (D–A)-type host materials, MR materials utilize the multiple resonance effect between electron-deficient and electron-rich atoms within a rigid, planar fused-ring structure [14]. Consequently, the highest occupied molecular orbital (HOMO) and lowest unoccupied molecular orbital (LUMO) are alternately localized on different atoms within the same planar framework, giving rise to short-range charge-transfer (SRCT) character. This orbital distribution pattern reduces the exchange interaction and thereby enables a small singlet–triplet energy gap (ΔE_ST_). The alternating arrangement of B/N or B/O atoms also limits the formation of a continuous π-conjugation pathway across the core. In particular, oxygen incorporation has been reported to restrict HOMO conjugation more strongly than nitrogen incorporation, resulting in a shift toward shorter-wavelength emission [15]. Accordingly, 5,9-dioxa-13b-boranaphtho[3,2,1-de]anthracene (DOBNA), which contains oxygen instead of nitrogen as the heteroatom, was selected as the backbone in this study. Moreover, the DOBNA core has a rigid fused-ring structure containing C–B and C–O bonds and lacks the vulnerable C–P bonds associated with phosphine oxide-based hosts, offering a potential advantage in chemical stability.

However, the low molecular weight (below 500) and relatively high crystallinity of DOBNA make it difficult to form stable thin films. To address this limitation, TDBA, a DOBNA derivative bearing bulky tert-butyl groups on the backbone, was reported to improve thin-film stability [16].

Simultaneously achieving high color purity and high efficiency remains a key challenge in blue OLEDs. Multiple-resonance thermally activated delayed fluorescence (MR-TADF) emitters exhibit high color purity owing to their narrow full width at half maximum (FWHM) arising from rigid fused-ring structures and can utilize triplet excitons for emission, making them promising emissive materials that have been investigated for high-efficiency blue OLEDs [14,17]. DABNA-1, reported by Hatakeyama et al., exhibited blue emission with a maximum external quantum efficiency (EQE_max_) of 13.5% and Commission Internationale de l’Eclairage (CIE) coordinates of (0.13, 0.09) [14], and the subsequently reported ν-DABNA exhibited a maximum electroluminescence wavelength (EL_max_) of 469 nm, an FWHM of 18 nm, and an EQE_max_ of 34.4% in a device doped into the DOBNA-based host DOBNA-OAr [17].

An EQE_max_ of 27.5% was reported for a hyperfluorescence device using DPEPO as the host and co-doped with a TADF sensitizer, and quenching associated with excimer formation in ν-DABNA was identified in that study as a factor limiting device performance [18]. Among TDBA-based hosts, TDBA-Si and mTDBA-2Ge, incorporating tetraphenylsilyl and tetraphenylgermyl groups, respectively, exhibited EQE_max_ values of 36.2% and 24.4%, respectively, in ν-DABNA devices [16,19]. These studies focused on controlling intermolecular packing using electronically neutral and bulky substituents.

In this study, two new electron-accepting host materials, TDBA-Py and TDBA-dPy, were designed and synthesized by introducing one and two electron-withdrawing pyridine moieties, respectively, into TDBA-Ph, a known structure in which a phenyl group is attached to TDBA. In this study, we investigate the effects of the strong dipole–dipole interactions of the pyridine nitrogen atoms on the molecular packing and thermal stability in thin films and further investigate the effect of increasing the number of pyridine substituents on the performance of the resulting hosts [16].

## 2. Experimental

### 2.1. Synthesis

#### 2.1.1. 4,4′-((2,5-Dibromo-1,3-phenylene)bis(oxy))bis(tert-butylbenzene) (**1**)

In a 3-neck round-bottom flask under a N_2_ atmosphere, 2,5-dibromo-1,3-difluorobenzene (10.0 g, 36.8 mmol), 4-(tert-butyl)phenol (16.6 g, 110 mmol), and potassium carbonate (K_2_CO_3_, 15.3 g, 110 mmol) were dissolved in anhydrous N-methyl-2-pyrrolidone (NMP, 45 mL). The mixture was stirred at 170 °C for 20 h. After the reaction was finished, the product was extracted using dichloromethane (DCM) and water three times. The organic phase was dried with anhydrous magnesium sulfate (MgSO_4_), filtered, and the solvent was evaporated. The crude product was purified by reprecipitating with methanol, obtaining a white solid (yield: 61%). ^1^H NMR (400 MHz, CDCl_3_): δ 7.42–7.36 (m, 4H), 7.00–6.93 (m, 4H), 6.72 (s, 2H), 1.33 (s, 18H) ppm.

#### 2.1.2. 7-Bromo-2,12-di-tert-butyl-5,9-dioxa-13b-boranaphtho[3,2,1-de]anthracene (**2**)

Under a N_2_ atmosphere, 4,4′-((2,5-dibromo-1,3-phenylene)bis(oxy))bis(tert-butylbenzene) (5.00 g, 11.0 mmol) was dissolved in anhydrous m-xylene (60 mL). After stirring for 30 min, n-butyllithium solution (n-BuLi, 2.5 M solution in hexane, 5.30 mL) was added dropwise at −50 °C. Then, the mixture was stirred at room temperature for 1 h. Then, boron tribromide (BBr_3_, 1.57 mL, 16.5 mmol) was slowly added at −50 °C, and the reaction mixture was then stirred at room temperature for 30 min, followed by a 1 h reaction at 45 °C. Then N,N-diisopropylethylamine (DIPEA, 3.84 mL, 22.1 mmol) was added dropwise at −15 °C. The mixture was stirred at room temperature for 30 min and then stirred at 140 °C for 20 h. After the reaction was completed, the mixture was cooled to room temperature and extracted with DCM and water three times. The organic phase was dried with anhydrous MgSO_4_ and filtered. The solvent was condensed by evaporation under vacuum, and the crude product was purified by silica gel column chromatography (DCM: n-hexane = 1:4 *v*/*v*) to afford a white solid (yield: 45%). ^1^H NMR (400 MHz, DMSO-d_6_): δ 8.45 (d, J = 2.4 Hz, 2H), 7.74–7.66 (m, 2H), 7.37 (d, J = 8.7 Hz, 2H), 7.33 (s, 2H), 1.38 (s, 18H) ppm.

#### 2.1.3. 2,12-Di-tert-butyl-7-(4,4,5,5-tetramethyl-1,3,2-dioxaborolan-2-yl)-5,9-dioxa-13b-boranaphtho[3,2,1-de]anthracene (**3**)

Under a N_2_ atmosphere, 7-bromo-2,12-di-tert-butyl-5,9-dioxa-13b-boranaphtho[3,2,1-de]anthracene (10.0 g, 21.7 mmol), bis(pinacolato)diboron (6.06 g, 23.9 mmol), potassium acetate (KOAc, 6.38 g, 65.0 mmol), and [1,1′-Bis(diphenylphosphino)ferrocene]dichloropalladium(II) (Pd(dppf)Cl_2_, 0.793 g, 1.08 mmol) were mixed in 1,4-dioxane (300 mL). The mixture was stirred at reflux for 2 h, with the reaction progress monitored by thin-layer chromatography (TLC). After the reaction was completed, the mixture was cooled to room temperature and extracted with DCM and water three times. The organic phase was dried with anhydrous MgSO_4_ and filtered. The solvent was evaporated under vacuum, and the crude product was purified by silica gel column chromatography (ethyl acetate (EA): n-hexane = 1:4 *v*/*v*) to afford a white solid (yield: 62%). ^1^H NMR (400 MHz, CDCl_3_): δ 8.74 (d, J = 2.5 Hz, 2H), 7.78–7.74 (m, 2H), 7.64 (s, 2H), 7.48 (d, J = 8.8 Hz, 2H), 1.47 (s, 18H), 1.39 (s, 12H) ppm.

#### 2.1.4. 3-(3-Bromophenyl)pyridine (**4**)

1-bromo-3-iodobenzene (5.00 g, 17.8 mmol), 3-(4,4,5,5-tetramethyl-1,3,2-dioxaborolan-2-yl)pyridine (3.63 g, 17.7 mmol), K_2_CO_3_ (5.50 g, 40.0 mmol), tetrakis(triphenylphosphine)palladium(0) (Pd(PPh_3_)_4_, 0.408 g, 0.354 mmol), ethanol (16.5 mL), distilled water (33 mL), and toluene (50 mL) were mixed under a nitrogen atmosphere. The mixture was stirred under reflux for 24 h. After the reaction was completed, the mixture was cooled to room temperature and extracted with toluene and water three times. The organic phase was dried with anhydrous MgSO_4_ and filtered. The solvent was evaporated under vacuum, and the crude product was purified by silica gel column chromatography (EA: n-hexane = 3:7 *v*/*v*) to afford yellow oil (yield: 85%). ^1^H NMR (400 MHz, CDCl_3_): δ 8.75 (d, J = 1.9 Hz, 1H), 8.59–8.54 (m, 1H), 7.80–7.73 (m, 1H), 7.65 (t, J = 1.8 Hz, 1H), 7.50–7.41 (m, 2H), 7.33–7.25 (m, 2H) ppm.

#### 2.1.5. 3-(3-(4,4,5,5-Tetramethyl-1,3,2-dioxaborolan-2-yl)phenyl)pyridine (**5**)

Under a N_2_ atmosphere, 3-(3-bromophenyl)pyridine (1.00 g, 4.27 mmol), bis(pinacolato)diboron (3.25 g, 12.8 mmol), KOAc (1.01 g, 10.3 mmol), and Pd(dppf)Cl_2_ (0.625 g, 0.854 mmol) were mixed in 1,4-dioxane (30 mL). The mixture was refluxed for 3 h, with the reaction progress monitored by TLC. After the reaction was completed, the mixture was cooled to room temperature and extracted with EA and water three times. The organic phase was dried with anhydrous MgSO_4_ and filtered. The solvent was evaporated under vacuum, and the crude product was purified by silica gel column chromatography (EA: n-hexane = 1:4 *v*/*v*) to afford a colorless solid (yield: 53%). ^1^H NMR (400 MHz, CDCl_3_): δ 8.88–8.83 (m, 1H), 8.59–8.54 (m, 1H), 8.01 (s, 1H), 7.93–7.87 (m, 1H), 7.86–7.81 (m, 1H), 7.70–7.64 (m, 1H), 7.51–7.44 (m, 1H), 7.36–7.30 (m, 1H), 1.35 (s, 12H) ppm.

#### 2.1.6. 3-(3-(2,12-Di-tert-butyl-5,9-dioxa-13b-boranaphtho[3,2,1-de]anthracen-7-yl)phenyl)pyridine (TDBA-Py)

Under a N_2_ atmosphere, 7-bromo-2,12-di-tert-butyl-5,9-dioxa-13b-boranaphtho[3,2,1-de]anthracene (0.50 g, 1.08 mmol), 3-(3-(4,4,5,5-tetramethyl-1,3,2-dioxaborolan-2-yl)phenyl)pyridine (0.366 g, 1.30 mmol), K_2_CO_3_ (0.449 g, 3.25 mmol), and Pd(PPh_3_)_4_ (0.125 g, 0.108 mmol) were dissolved in toluene (20 mL) and distilled water (10 mL). The mixture was stirred under reflux for 2 h, with the reaction progress monitored by TLC. After the reaction was completed, the mixture was cooled to room temperature and extracted with EA and water three times. The organic phase was dried with anhydrous MgSO_4_ and filtered. The solvent was evaporated under vacuum, and the crude product was purified by silica gel column chromatography (EA: n-hexane = 1:9 *v*/*v*) to afford a colorless solid (yield: 27%). ^1^H NMR (400 MHz, CD_2_Cl_2_): δ 8.91–8.85 (m, 1H), 8.73 (d, J = 2.5 Hz, 2H), 8.61–8.54 (m, 1H), 8.03–7.97 (m, 1H), 7.94 (d, J = 1.6 Hz, 1H), 7.83–7.72 (m, 3H), 7.67–7.57 (m, 2H), 7.51–7.45 (m, 4H), 7.44–7.38 (m, 1H), 1.47 (s, 18H) ppm. ^13^C NMR (101 MHz, CDCl_3_): δ 158.61, 158.16, 147.94, 147.87, 146.25, 145.24, 141.93, 138.20, 137.04, 135.15, 130.66, 130.29, 129.86, 127.65, 126.87, 126.57, 124.08, 123.29, 117.95, 107.39, 34.69, 31.75 ppm.

GC-HRMS (*m*/*z*): [M+] calc’d for C_37_H_34_BNO_2_, 535.48; found, 536.28; Elemental analysis calc’d (%) for C_37_H_34_BNO_2_: C 82.99, H 6.40, N 2.62, O 5.98; found: C 82.46, H 6.47, N 2.99, O 5.94%.

#### 2.1.7. 3,3′-(5-Bromo-1,3-phenylene)dipyridine (**6**)

Under a N_2_ atmosphere, 1,3,5-tribromobenzene (1.00 g, 3.18 mmol), 3-(4,4,5,5-tetramethyl-1,3,2-dioxaborolan-2-yl)pyridine (1.43 g, 6.99 mmol), K_2_CO_3_ (2 M aqueous solution, 5.31 mL), and Pd(PPh_3_)_4_ (0.219 g, 0.191 mmol) were mixed with toluene (30 mL). The mixture was refluxed for 20 h and was monitored by TLC. After the reaction was completed, the mixture was cooled to room temperature and extracted with chloroform and water three times. The organic phase was dried with anhydrous MgSO_4_ and filtered. The solvent was evaporated under vacuum, and the crude product was purified by silica gel column chromatography (chloroform: tetrahydrofuran (THF) = 1:3 *v*/*v*) to afford a white solid (yield: 14%). ^1^H NMR (400 MHz, CDCl_3_): δ 8.88 (s, 2H), 8.67 (d, J = 4.0 Hz, 2H), 7.95–7.87 (m, 2H), 7.76 (d, J = 1.6 Hz, 2H), 7.68 (t, J = 1.5 Hz, 1H), 7.46–7.37 (m, 2H) ppm.

#### 2.1.8. 3,3′-(5-(2,12-Di-tert-butyl-5,9-dioxa-13b-boranaphtho[3,2,1-de]anthracen-7-yl)-1,3-phenylene)dipyridine (TDBA-dPy)

Under a N_2_ atmosphere, 2,12-di-tert-butyl-7-(4,4,5,5-tetramethyl-1,3,2-dioxaborolan-2-yl)-5,9-dioxa-13b-boranaphtho[3,2,1-de]anthracene (2.00 g, 3.94 mmol), 3,3′-(5-bromo-1,3-phenylene)dipyridine (0.857 g, 2.75 mmol), K_2_CO_3_ (2 M aqueous solution, 5.90 mL), and Pd(PPh_3_)_4_ (0.455 g, 0.394 mmol) were mixed with toluene (40 mL) and distilled water (10 mL). The mixture was refluxed for 20 h, and the reaction was monitored using TLC. After the reaction was completed, the mixture was cooled to room temperature and extracted with chloroform and water three times. The organic phase was dried with anhydrous MgSO_4_ and filtered. The solvent was evaporated under vacuum, and the crude product was purified by silica gel column chromatography (THF: n-hexane = 1:1 *v*/*v*) to afford a white solid (yield: 11%). ^1^H NMR (400 MHz, CDCl_3_): δ 8.97 (s, 2H), 8.71 (d, J = 2.4 Hz, 2H), 8.68 (d, J = 3.8 Hz, 2H), 8.05–8.02 (m, 2H), 7.94 (d, J = 1.6 Hz, 2H), 7.78 (t, J = 1.6 Hz, 1H), 7.75–7.71 (m, 2H), 7.50 (s, 2H), 7.49–7.45 (m, 4H), 1.49 (s, 18H) ppm. ^13^C NMR (101 MHz, CDCl_3_): δ 158.30, 158.04, 148.10, 147.81, 145.32, 144.49, 143.25, 139.12, 136.74, 135.38, 130.12, 129.39, 126.50, 125.38, 124.91, 124.23, 117.70, 107.64, 34.67, 31.81 ppm.

GC-HRMS (*m*/*z*): [M+] calc’d for C_42_H_37_BN_2_O_2_, 612.57; found, 613.30; elemental analysis calc’d (%) for C_42_H_37_BN_2_O_2_: C 82.35, H 6.09, N 4.57, O 5.22; found: C 79.99, H 6.40, N 3.97, O 4.97%.

## 3. Results and Discussion

### 3.1. Molecular Design, Synthesis, and Characterization

The TDBA moiety, a DOBNA-based MR core, features a rigid fused framework containing a central boron atom and oxygen atoms. This structural rigidity restricts molecular vibrations and suppresses nonradiative decay pathways, making it advantageous for achieving high thermal stability and a high triplet energy level [20,21]. In our previous studies, we reported host materials bearing various substituents on the TDBA moiety, including TDBA-Ph, TDBA-Si, and TDBA-Ge [16,19]. Among these materials, the phenyl group in TDBA-Ph was introduced as a steric spacer to suppress intermolecular π–π stacking of the highly planar TDBA cores. As an electronically neutral substituent, the phenyl group offers the advantage of preserving the HOMO and LUMO distributions and emission wavelength of TDBA. However, because of this electronic neutrality, the phenyl group alone has limited ability to tune the charge-transport properties of the host.

**Figure 1:**
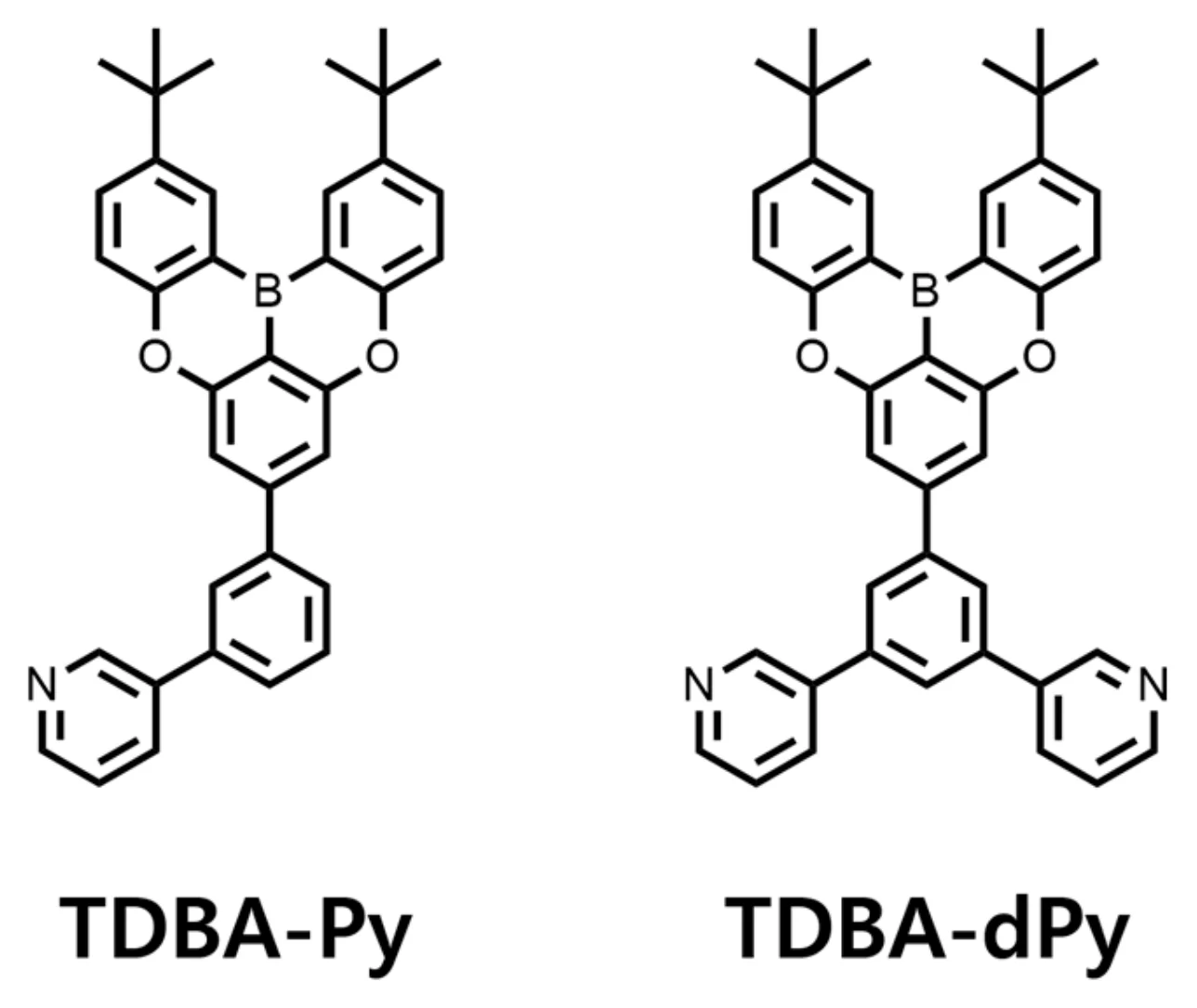
Chemical structures of TDBA-Py and TDBA-dPy.

**Scheme 1:**
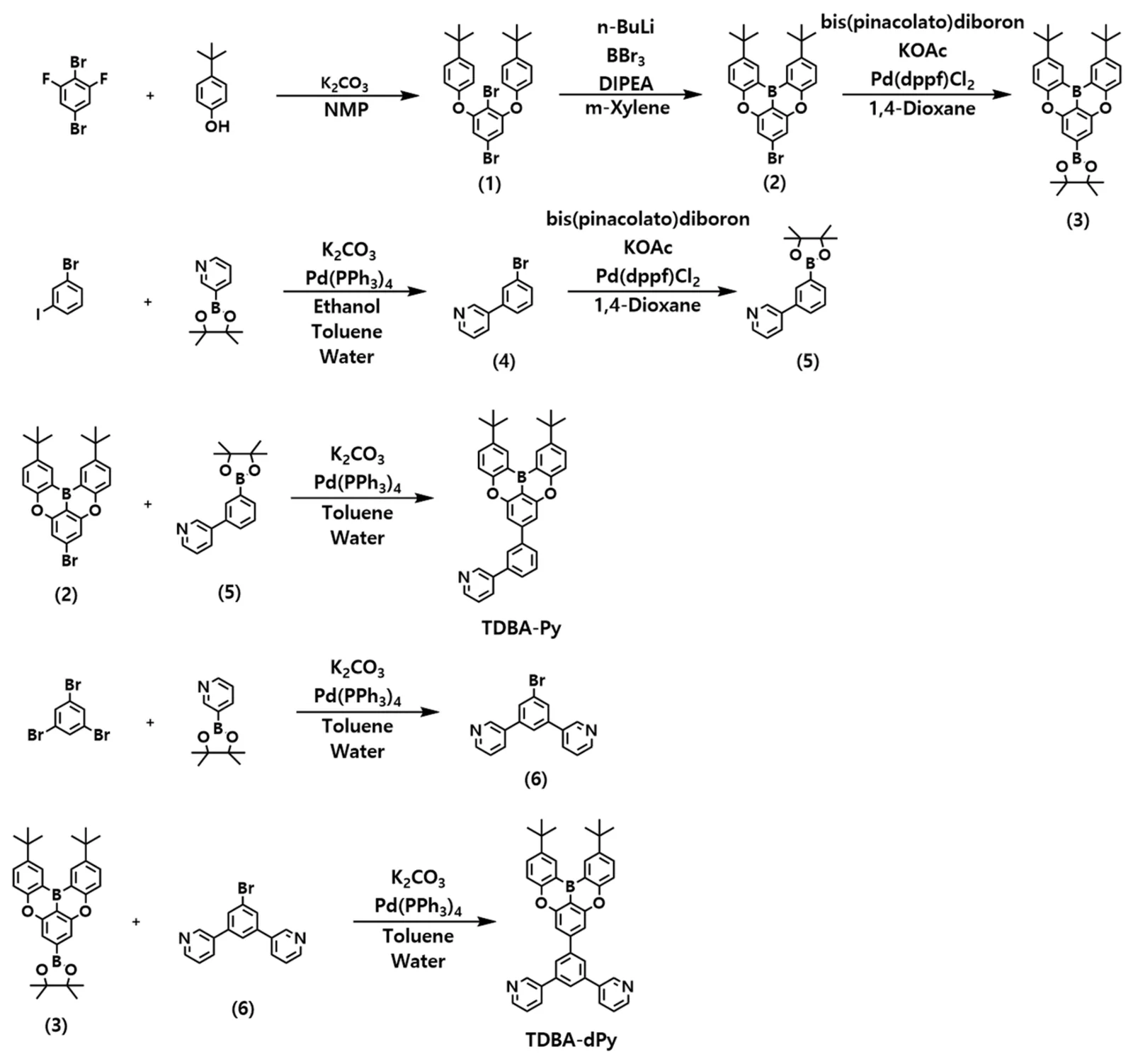
Synthetic routes of TDBA-Py and TDBA-dPy.

To address this limitation, we synthesized two new electron-accepting blue host materials, TDBA-Py and TDBA-dPy, by attaching one and two pyridine groups at the meta positions of the phenyl group in TDBA-Ph (Figure 1). The introduction of pyridine as an electron-withdrawing group was expected to impart electron-accepting character to the host and to lower the LUMO level relative to that of TDBA-Ph, thereby reducing the electron-injection barrier [16]. The polarity of the pyridine groups may strengthen intermolecular interactions and thereby improve thermal stability [22,23,24]. The pyridine groups were introduced at the meta positions of the phenyl groups to suppress the extension of intramolecular conjugation. When the pyridine group is attached at the para position, the conjugation of the entire molecule can be extended. Such an increase in conjugation could narrow the band gap and redshift the emission wavelength, potentially limiting the material’s suitability as a host. In contrast, attaching pyridine groups at the meta positions can limit the extension of conjugation and prevent a red shift in the emission wavelength. This design may preserve the optical properties and high T_1_ of TDBA and retain the advantages conferred by pyridine [25]. During the synthesis of TDBA-dPy, the coupling reaction between bromide-containing compound (**2**) and the borylated pyridine-substituted derivative gave an extremely low yield. To overcome this problem, compound (**2**) was borylated to afford compound (**3**), which was subsequently coupled with the bromide-containing pyridine substituent (**6**) to give TDBA-dPy (Scheme 1). The structures of the final products were confirmed by ^1^H NMR spectroscopy and mass spectrometry (Figures S1–S12).

### 3.2. Theoretical Calculations

To investigate the bonding characteristics between the TDBA moiety and the side group, density functional theory (DFT) calculations were performed. All calculations were performed using the ORCA program. Geometry optimizations and the evaluation of the lowest excited singlet (S_1_) and triplet (T_1_) states were carried out at the B3LYP-D3/def2-TZVPP level.

**Figure 2:**
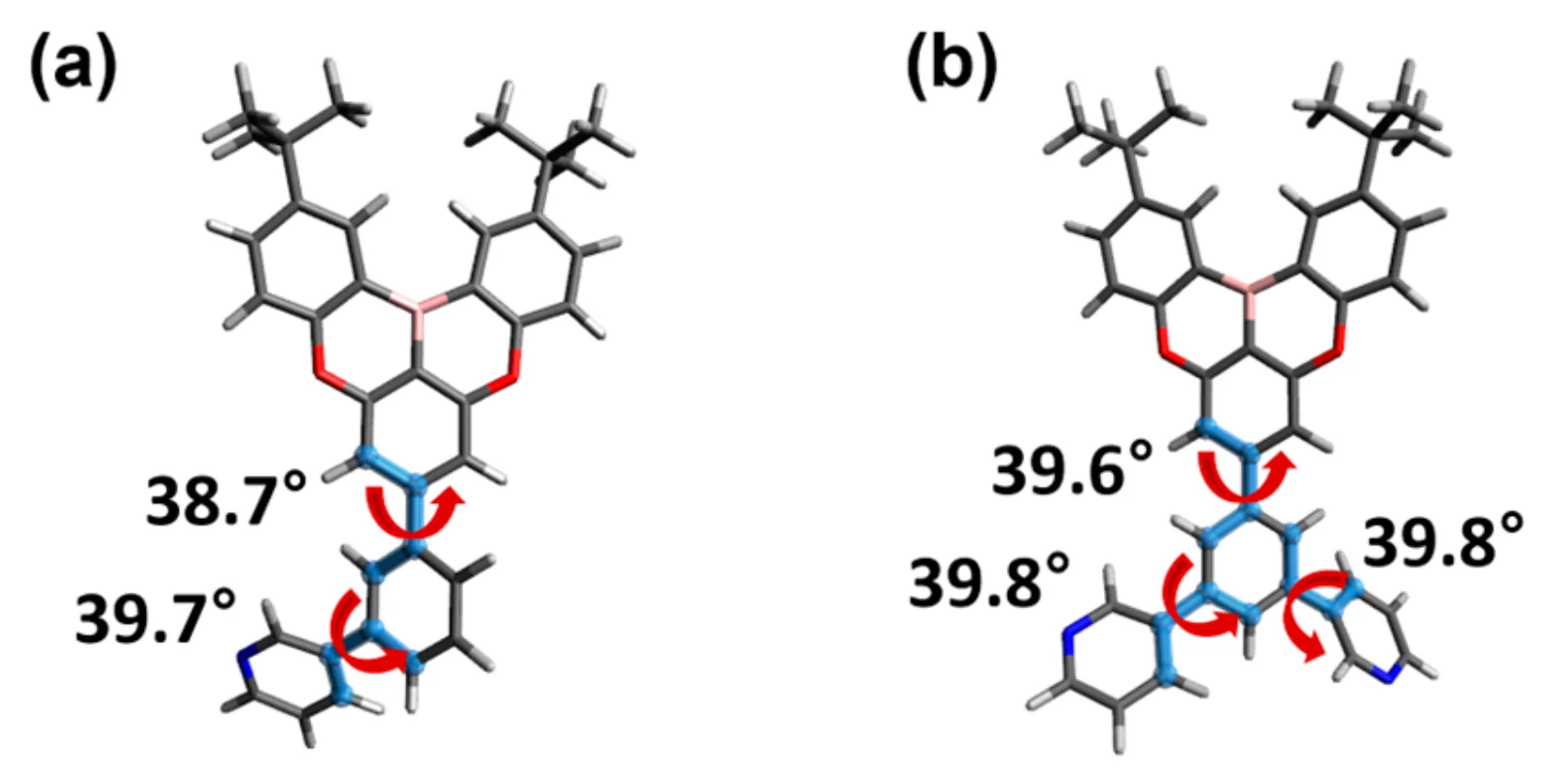
Dihedral angles between the core and peripheral rings calculated by DFT theory: (**a**) TDBA-Py; (**b**) TDBA-dPy.

In the optimized ground-state geometries, the dihedral angles between the TDBA moiety and the introduced side group and between the phenyl ring and the attached pyridine were similar for both TDBA-Py and TDBA-dPy, ranging from 38.7 to 39.8° (Figure 2). This indicates that the introduction of pyridine substituents does not cause a significant difference in the steric twist at the single-molecule level. Therefore, the main emission of both materials is expected to originate from the TDBA moiety.

**Figure 3:**
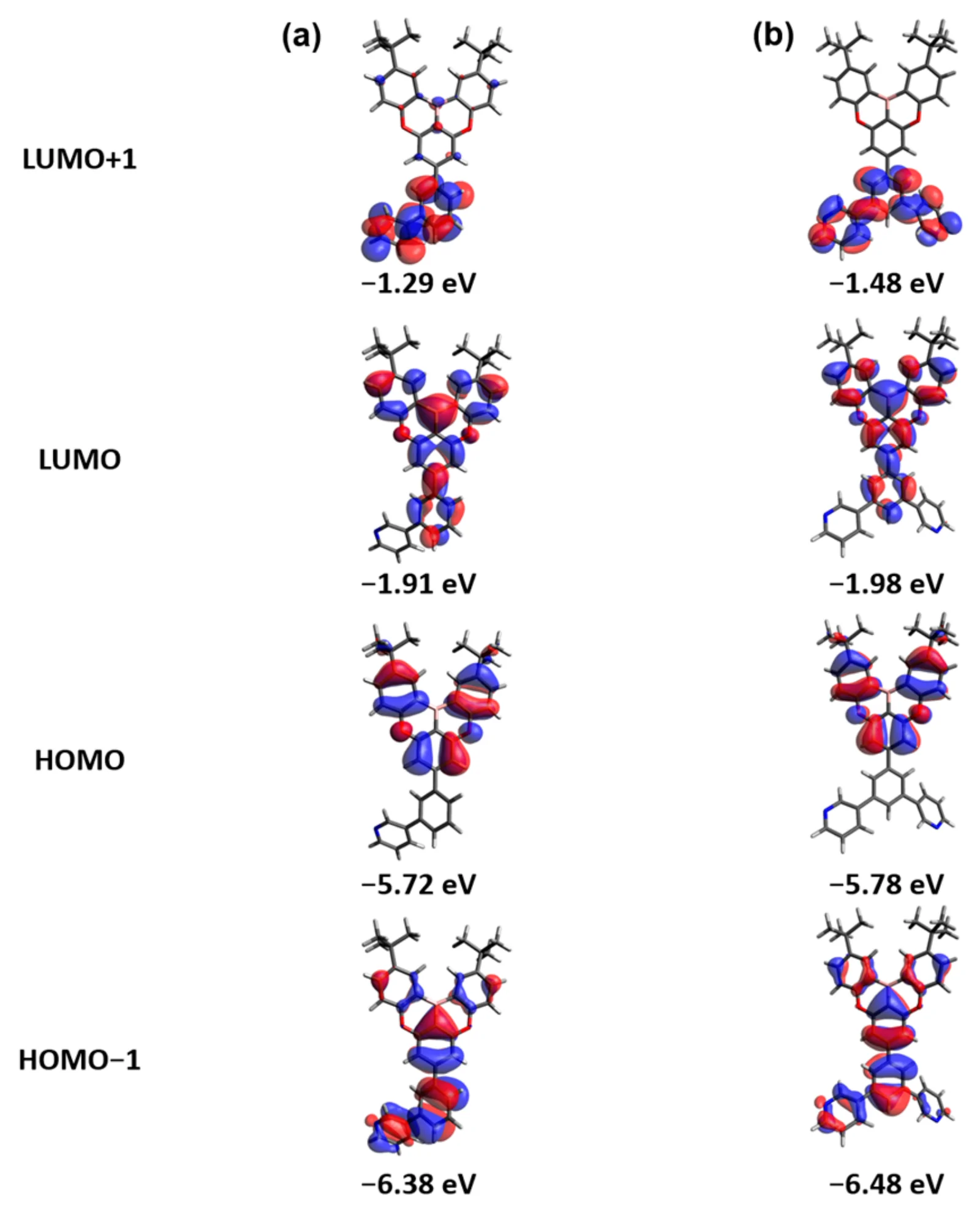
Molecular orbitals and energy levels of molecules calculated by DFT theory: (**a**) TDBA-Py; (**b**) TDBA-dPy.

The electron densities of the HOMO and LUMO of TDBA-Py and TDBA-dPy were mainly distributed on the TDBA core, and this distribution pattern was maintained as the number of pyridine substituents increased (Figure 3). However, the electron density of the HOMO−1 extended over both the TDBA and pyridine moieties, and that of the LUMO+1 was located on the pyridine moiety, indicating a weak electron-accepting character induced by pyridine. The calculated HOMO energy levels of TDBA-Py and TDBA-dPy were −5.72 and −5.78 eV, and their LUMO energy levels were −1.91 and −1.98 eV, indicating similar energy levels for the two compounds. The molecular calculations yielded an S_1_ energy of 3.27 eV for both TDBA-Py and TDBA-dPy and T_1_ energies of 2.78 and 2.77 eV, respectively. The resulting ΔE_ST_ values were 0.49 and 0.50 eV, which were similar for the two materials.

### 3.3. Photophysical and Electrochemical Properties

**Figure 4:**
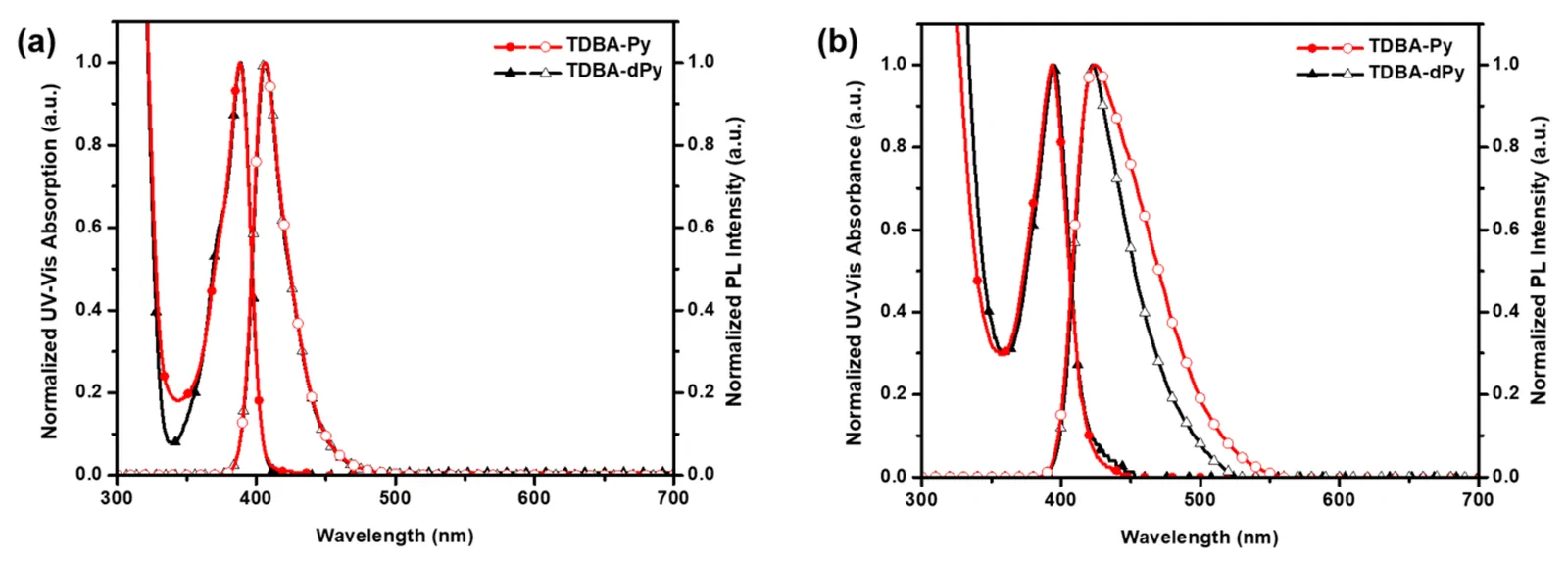
Optical properties of TDBA-Py and TDBA-dPy: (**a**) in solution state; (**b**) in thin-film state.

To investigate the photophysical characteristics of newly synthesized TDBA-based host materials, TDBA-Py and TDBA-dPy, ultraviolet–visible (UV-vis) absorption and photoluminescence (PL) spectra were measured in both toluene solution and vacuum-deposited film states (Figure 4). Table 1 summarizes the photophysical characteristics of the two TDBA-based host materials. In solution, both compounds exhibited a maximum absorption wavelength (UV_max_) of 388 nm. The maximum photoluminescence wavelength (PL_max_) values of TDBA-Py and TDBA-dPy were also similar, at 407 and 406 nm. Unsubstituted TDBA was reported to exhibit a UV_max_ of 383 nm, a PL_max_ of 403 nm, and an FWHM of 27 nm under the same conditions [16]. Compared with TDBA, the UV_max_ and PL_max_ of the two materials were redshifted by 5 nm and 3–4 nm, respectively, and the FWHM remained identical at 27 nm. These results indicate that emission originates predominantly from the TDBA core and that pyridine incorporation does not substantially affect the optical properties [26]. In solution, TDBA-Py and TDBA-dPy exhibited small Stokes shifts of 19 and 18 nm, both below 20 nm. This can be attributed to the rigid fused-ring structure of the TDBA core, which minimizes structural relaxation in the excited state [15,27].

In the film state, the UV_max_ values of TDBA-Py and TDBA-dPy were 393 and 395 nm, and their PL_max_ values were 424 and 422 nm. As in the solution state, the two materials showed similar UV_max_ and PL_max_ values. For both materials, UV_max_ and PL_max_ shifted to longer wavelengths in the film state relative to solution, but their emission remained in the blue region. As a result, the meta-substituted pyridine barely changed the absorption and emission wavelengths in both solution and thin films. However, a clear difference was observed in the FWHM in the film state: TDBA-Py and TDBA-dPy exhibited FWHM values of 64 and 48 nm. The FWHM of TDBA-dPy was 16 nm narrower than that of TDBA-Py. In general, when molecules transition from the solution state to the film state, the reduced intermolecular distance induces intermolecular interactions that stabilize the excited state and narrow the band gap, resulting in a redshifted emission. The increased energetic disorder arising from diverse local molecular environments broadens the FWHM. The narrower FWHM of TDBA-dPy relative to TDBA-Py may be attributed to its bulky dipyridylphenyl substituent, which is structurally similar to m-terphenyl, a moiety known to suppress intermolecular π–π interactions. This steric bulk may more effectively limit aggregation-induced spectral broadening in the film state [28,29].

In solution, TDBA-Py and TDBA-dPy exhibited similar photoluminescence quantum yield (PLQY) values of 50% and 47%. In the film state, the PLQY values of TDBA-Py and TDBA-dPy were 37% and 23%, indicating a decrease with increasing pyridine substitution that was not observed in solution. This may be attributed to the molecular structures of TDBA-Py and TDBA-dPy, which permit the formation of weak intermolecular hydrogen bonds involving the pyridine groups as molecules approach one another in the film state [30,31]. In particular, the two pyridine units in TDBA-dPy act as bulky groups that suppress intermolecular packing, allowing a narrow FWHM to be maintained. In addition, the lower PLQY of TDBA-dPy may be attributed to its greater number of pyridine groups, which could enable more diverse hydrogen-bonding interactions and activate nonradiative decay pathways, resulting in emission quenching.

To determine the S_1_ and T_1_ energy levels of TDBA-Py and TDBA-dPy, PL spectra of their thin films were measured at room temperature and 77 K (Figure S13). The S_1_ and T_1_ energy levels were estimated from the onsets of the spectra measured at room temperature and 77 K. In the film state, TDBA-Py and TDBA-dPy exhibited S_1_ energies of 3.15 and 3.10 eV and T_1_ energies of 2.87 and 2.76 eV. The introduction of a second pyridine unit lowered the T_1_ level by 0.11 eV. Both materials retained high T_1_ energy levels above 2.7 eV, indicating their suitability as hosts for blue-emitting devices (Table 2). The experimentally determined ΔE_ST_ values were 0.28 and 0.34 eV, which were 0.16–0.21 eV lower than the calculated values (0.49, 0.50 eV). For MR frameworks, time-dependent DFT (TD-DFT) calculations are known to predict S_1_ energies and ΔE_ST_ values higher than the experimental values [32,33].

The HOMO levels for TDBA-Py and TDBA-dPy were experimentally determined using a photoelectron spectrometer (AC-2), and the LUMO levels were calculated from the HOMO level and optical band gap. The optical band gap was derived from the (αhν)^2^ versus (hν) plot by determining the absorbance edge, where α, h, and ν represent absorbance, Planck’s constant, and the frequency of light, respectively. The HOMO levels of TDBA-Py and TDBA-dPy were −5.81 and −5.79 eV; LUMO levels were −2.89 and −2.86 eV; and the band gaps were 2.92 and 2.93 eV, respectively.

### 3.4. Thermal Properties

**Figure 5:**
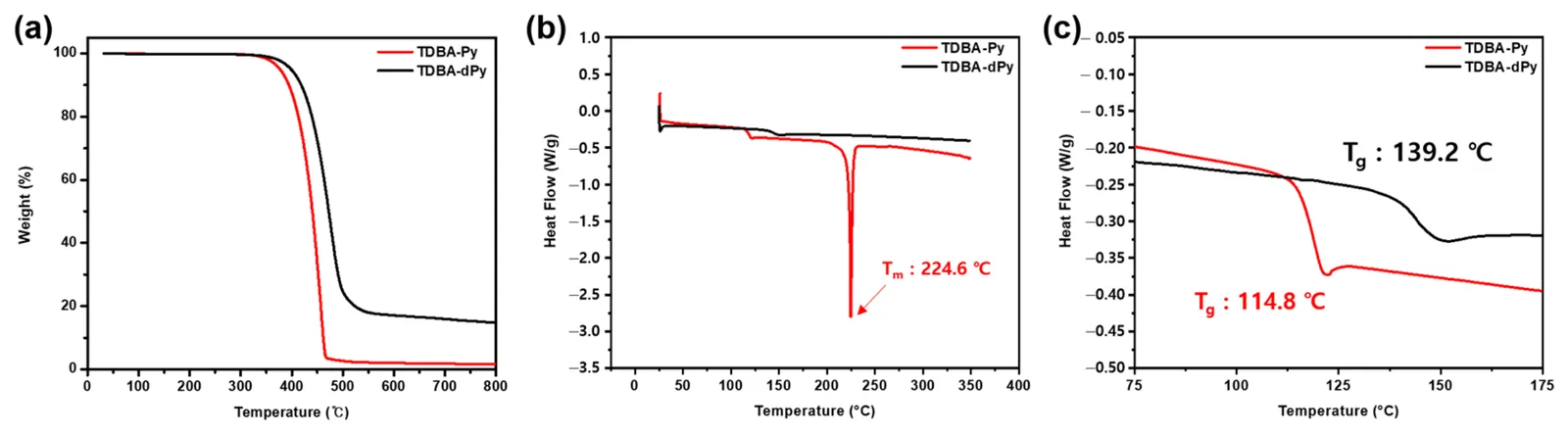
Thermal properties of TDBA-Py and TDBA-dPy: (**a**) TGA curves; (**b**) DSC curves; (**c**) enlarged DSC curves in the glass transition region.

The decomposition temperature (T_d_), glass transition temperature (T_g_), and melting temperature (T_m_) of TDBA-Py and TDBA-dPy were determined using thermogravimetric analysis (TGA) and differential scanning calorimetry (DSC), as illustrated in Figure 5. The T_d_ values corresponding to 5% weight loss for TDBA-Py and TDBA-dPy were 376 °C and 398 °C. Compared to the T_d_ value of TDBA-Ph (330 °C), the two molecules showed increased thermal stability. A similar trend was observed in the T_g_ values. TDBA-Ph has been reported to exhibit a T_g_ of 62 °C. Such a low T_g_ may allow the film to crystallize under Joule heating during device operation, which could degrade its morphological stability [34,35]. In contrast, TDBA-Py and TDBA-dPy exhibited T_g_ values of 115 °C and 139 °C, corresponding to increases of 53 °C and 77 °C relative to TDBA-Ph. These values exceed the typical operating temperature range of OLEDs. The higher T_d_ and T_g_ values of TDBA-Py and TDBA-dPy relative to TDBA-Ph may be attributed to their increased molecular weights and stronger intermolecular interactions arising from the introduction of slightly polar pyridine groups in place of nonpolar phenyl groups.

A similar trend was observed for T_m_. TDBA-Py exhibited a T_m_ of 225 °C, whereas no melting transition was observed for TDBA-dPy within the investigated heating range of up to 350 °C. The absence of an observable melting transition for TDBA-dPy may be attributed to increased molecular rigidity and stronger intermolecular interactions associated with the additional pyridine group. These findings indicate that TDBA-Py and TDBA-dPy have higher thermal stability than TDBA-Ph and suggest that both materials can maintain stable device morphology within the operating temperature range of OLEDs [36]. Compared with commonly used hosts, the T_g_ values of TDBA-Py and TDBA-dPy (115, 139 °C) are higher than those of mCP (66 °C) and mCBP (93 °C) [37].

### 3.5. Electroluminescence (EL) Properties

To evaluate the host properties of the two materials, doped devices were fabricated. ν-DABNA was used as the dopant. Prior to device fabrication, the spectral overlap between the film PL spectra of the host materials and the solution UV spectrum of ν-DABNA was examined to confirm efficient energy transfer from the host to the dopant (Figure S14). Both materials showed substantial spectral overlap with the dopant, suggesting that Förster resonance energy transfer (FRET) from the hosts to the ν-DABNA dopant should occur [38]. Owing to its narrower FWHM of TDBA-dPy, however, the PL spectrum of TDBA-dPy overlapped less with the absorption peak of ν-DABNA than that of TDBA-Py. The T_1_ level of ν-DABNA (2.62 eV) is lower than those of both host materials, suggesting that Dexter energy transfer from the hosts to the dopant is energetically feasible [17,39].

**Figure 6:**
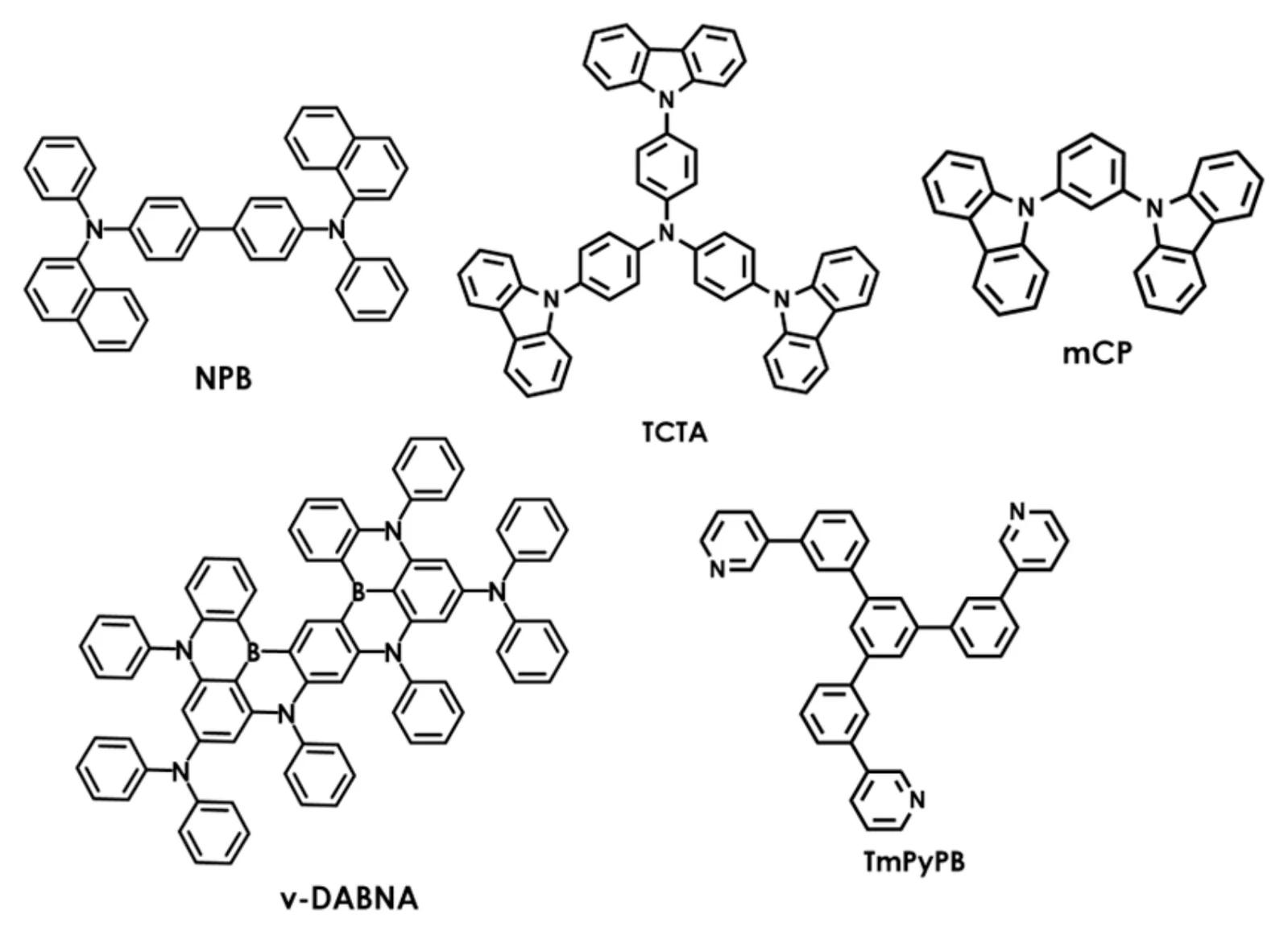
Molecular structures of the materials used in the doped devices.

**Figure 7:**
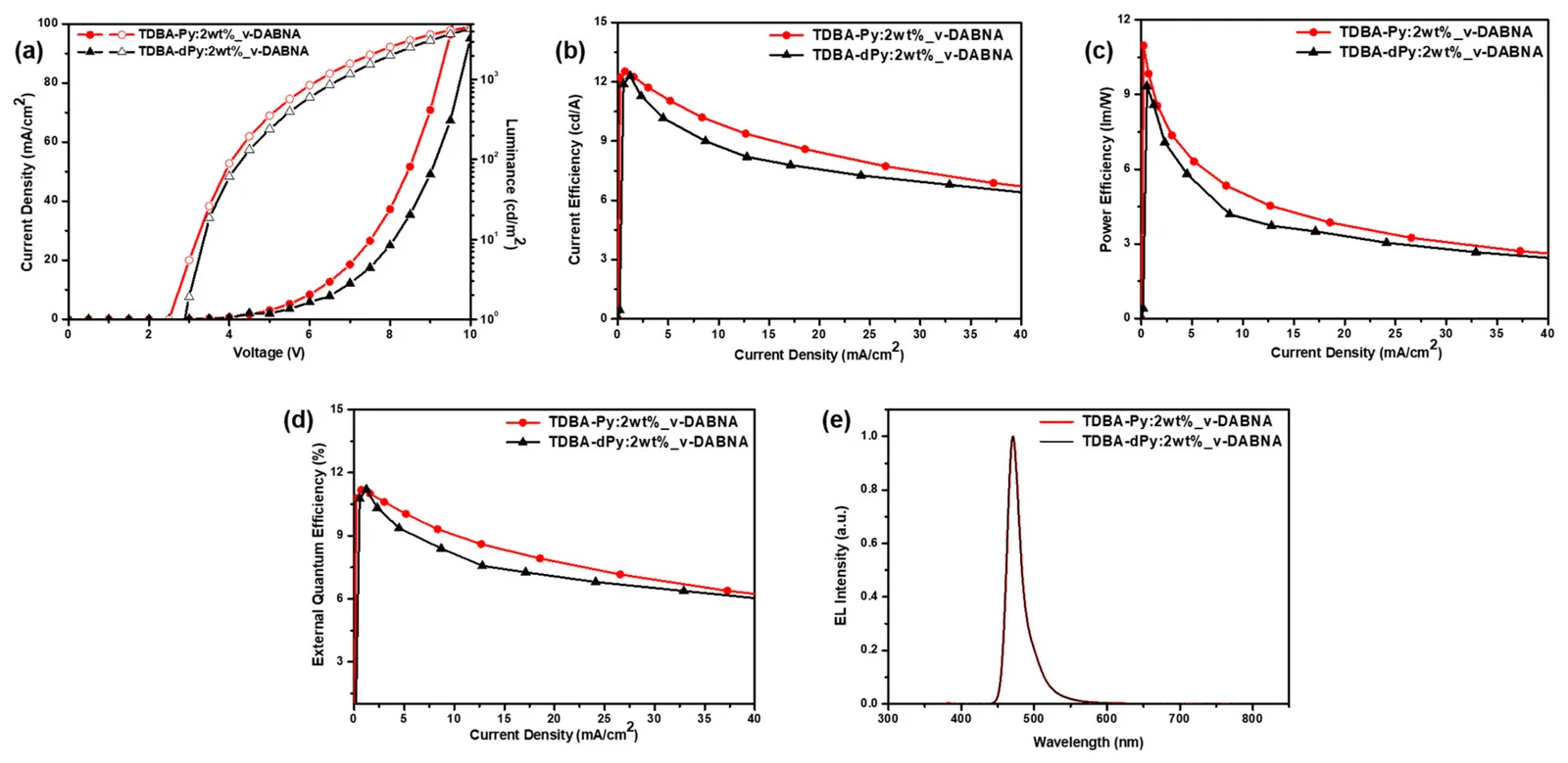
EL performance of doped device: (**a**) J–V–L curves; (**b**) CE curves; (**c**) PE curves; (**d**) EQE curves; (**e**) EL spectra.

The OLED device architecture was indium tin oxide (ITO)/N,N′-di(1-naphthyl)-N,N′-diphenyl-(1,1′-biphenyl)-4,4′-diamine (NPB) (40 nm)/TCTA (15 nm)/mCP (15 nm)/TDBA host: 2% ν-DABNA (20 nm)/TmPyPB (40 nm)/LiF (1 nm)/Al (200 nm). NPB and TCTA were used as the hole-injection and hole-transport layer materials, respectively. TDBA-Py and TDBA-dPy were used as the host materials, while ν-DABNA was used as the dopant material in the host–dopant system. TmPyPB was used as an electron-transporting and hole-blocking layer (Figure 6). The EL performance of the fabricated doped devices is summarized in Table 3 and shown in Figure 7.

**Figure 8:**
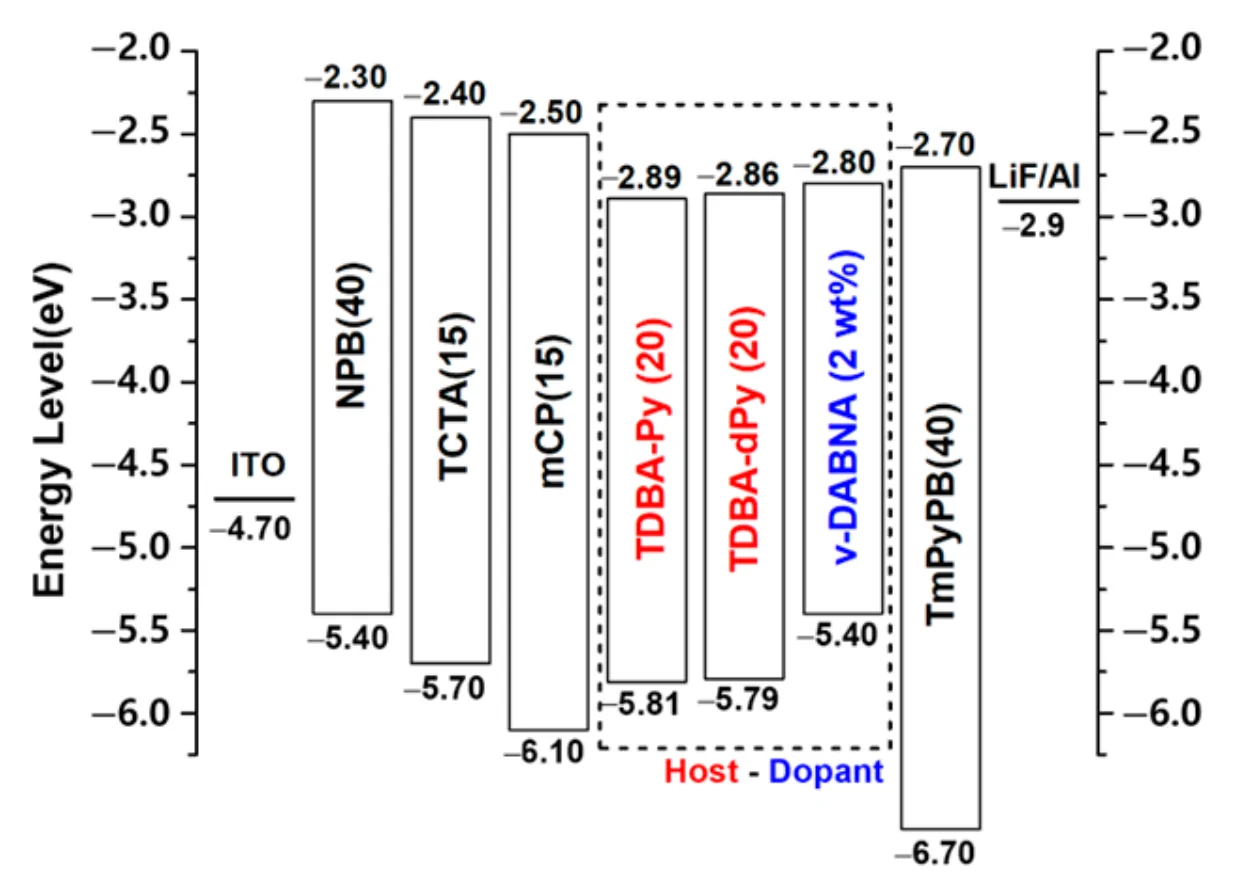
Band diagram of the doped device.

Devices employing TDBA-Py and TDBA-dPy as hosts exhibited turn-on voltages of 2.5 and 2.9 V, respectively, at a luminance of 1 cd m^−2^. The decrease in the turn-on voltages of the TDBA-Py- and TDBA-dPy-based devices compared with that of the reported TDBA-Ph-based device (3.1 V) is attributed to the lowered LUMO levels induced by the pyridine groups, which facilitate electron injection (Figure 8). To investigate the charge transport properties of TDBA-Py and TDBA-dPy, electron-only devices (EODs) and hole-only devices (HODs) were fabricated, and the charge carrier mobilities were determined using the space-charge-limited current (SCLC) method (Figure S15). In the range of 1.0–2.0 V (E^1/2^ = 447–632 V^1/2^ cm^−1/2^), the electron mobilities of TDBA-Py and TDBA-dPy were 1.9 × 10^−5^–1.5 × 10^−4^ cm^2^ V^−1^ s^−1^ and 4.0 × 10^−5^–3.6 × 10^−4^ cm^2^ V^−1^ s^−1^, respectively. In the same range, the hole mobilities of TDBA-Py and TDBA-dPy were 5.6 × 10^−8^–2.0 × 10^−7^ cm^2^ V^−1^ s^−1^ and 3.9 × 10^−8^–8.2 × 10^−8^ cm^2^ V^−1^ s^−1^, respectively, indicating that the electron mobility was more than 10^2^–10^3^ times higher than the hole mobility for both materials. Over this voltage range, TDBA-dPy, which incorporates two pyridine units, exhibited approximately twice the electron mobility of TDBA-Py, and TDBA-Py exhibited slightly higher hole mobility than TDBA-dPy.

The TDBA-Py-based device exhibited a maximum power efficiency (PE_max_) of 10.9 lm W^−1^, and its power efficiency (PE) at 500 cd m^−2^ was 6.65 lm W^−1^, which was higher than that of the TDBA-dPy-based device. The TDBA-dPy-based device showed a PE_max_ of 9.34 lm W^−1^. This may be attributable to the electron affinity of pyridine, which could cause the pyridine groups to act as charge traps during electron transport [40]. Consequently, the TDBA-dPy-based device exhibited a lower PE of 5.80 lm W^−1^ at 500 cd m^−2^. The PE_max_ values of both materials were higher than that of TDBA-Ph (9.01 lm W^−1^). Overall, the electron-accepting character of TDBA can be appropriately tuned by controlling the number of pyridine substituents, and the introduction of pyridine was confirmed to be an effective strategy for improving the efficiency from low current densities up to 500 cd m^−2^. The maximum current efficiency (CE_max_) values of the TDBA-Py- and TDBA-dPy-based devices were 12.5 and 12.3 cd A^−1^, and their current efficiency (CE) values at 500 cd m^−2^ were 11.0 and 10.2 cd A^−1^. These values are higher than the reported CE_max_ (10.8 cd A^−1^) and CE at 500 cd m^−2^ (9.80 cd A^−1^) of the TDBA-Ph-based device. Both materials exhibited higher current efficiencies than TDBA-Ph.

The TDBA-Py- and TDBA-dPy-based devices showed the same EQE_max_ of 11.2%, but their external quantum efficiency (EQE) values at 500 cd m^−2^ were 10.0% and 9.37%. The relatively lower EQE of the TDBA-dPy-based device may be attributed to its lower T_1_ level and the reduced overlap between its PL spectrum and the absorption spectrum of ν-DABNA compared with TDBA-Py, which could decrease FRET efficiency. Although TDBA-Py exhibited higher CE and PE than the reported TDBA-Ph-based device, TDBA-Ph showed higher EQE values, with an EQE_max_ of 12.9% and an EQE of 11.8% at 500 cd m^−2^. This is considered to result from the redshift of EL_max_ and the increase in FWHM relative to the TDBA-Ph-based device, which arises from the increased host polarity induced by the introduced pyridine and the resulting stronger interaction with the dopant [39]. Both devices showed the same EL_max_ of 471 nm and CIE coordinates of (0.12, 0.15), which are similar to the EL_max_ of ν-DABNA, suggesting that energy transfer from the host to the dopant occurred efficiently [16,17,39]. Devices with ν-DABNA doped into hosts such as mCBP, DCzPy, and DBFPO have been reported to exhibit turn-on voltages of 3.1–3.3 V, with EQE decreasing to 3.7–10.4% at 1000 cd m^−2^ [41,42]. Although the TDBA-Py device exhibited a lower EQE_max_ of 11.2% than these devices, it had a lower turn-on voltage of 2.5 V and maintained an EQE of 10.0% at 500 cd m^−2^.

## 4. Conclusions

In this study, new electron-accepting host materials, TDBA-Py and TDBA-dPy, were developed by introducing one and two pyridine units at the meta positions of the side phenyl ring of the TDBA framework, and the effects of the number of pyridine substituents on the physical and electrical properties and device performance of hosts were analyzed. By restricting the extension of intramolecular conjugation through a meta-linkage strategy, both materials exhibited PL_max_ values of 424 and 422 nm in the film state, maintaining emission in the blue region. In addition, the enhanced polar interactions arising from the introduction of pyridine improved the thermal stability, increasing the T_g_ values of TDBA-Py and TDBA-dPy to 115 and 139 °C, which are at least 1.8 times higher than that of TDBA-Ph (62 °C).

In the evaluation of blue-doped devices based on the ν-DABNA dopant, the LUMO levels stabilized by the pyridine groups lowered the electron-injection barrier and reduced the turn-on voltage of the devices. Owing to the improved charge balance in the EML resulting from its enhanced electron-injection properties, the TDBA-Py-based device achieved a PE_max_ of 10.9 lm W^−1^ and a CE_max_ of 12.5 cd A^−1^ and maintained a high CE of 11.0 cd A^−1^ at 500 cd m^−2^.

These findings demonstrate that introducing a single pyridine group through precise control of pyridine substitution on a rigid core is a promising molecular design strategy for blue hosts, improving electron injection and device efficiency without compromising high T_1_ or thermal stability.

## Figures and Tables

**Table 1:** The photophysical properties of TDBA-Py and TDBA-dPy.

	Solution ^a^			Film ^b^			PLQY (%) ^f^
	UV_max_ ^c^ (nm)	PL_max_ ^d^ (nm)	FWHM ^e^ (nm)	UV_max_ ^c^ (nm)	PL_max_ ^d^ (nm)	FWHM ^e^ (nm)	
TDBA-Py	388	407	27	393	424	64	50/37
TDBA-dPy	388	406	27	395	422	48	47/23

^a^ Toluene solution (concentration: 1 × 10^−5^ M). ^b^ In evaporated film state (thickness: 50 nm). ^c^ Maximum absorption wavelength. ^d^ Maximum photoluminescence wavelength. ^e^ Full width at half maximum. ^f^ Absolute photoluminescence quantum yield in solution (left) and film (right) states.

**Table 2:** Film-state S_1_, T_1_, and ΔE_ST_ values of TDBA-Py and TDBA-dPy determined from the onset wavelengths of prompt and delayed photoluminescence spectra.

	S_1_ (eV)	T_1_ (eV)	ΔE_ST_ (eV)
TDBA-Py	3.15	2.87	0.28
TDBA-dPy	3.10	2.76	0.34

**Table 3:** EL performance of doped devices.

	V_on_ (V) ^a^	L_max_ (cd m^−2^) ^b^	CE (cd A^−1^) ^c^ Max/@500 cd m^−2^	PE (lm W^−1^) ^d^ Max/@500 cd m^−2^	EQE (%) ^e^ Max/@500 cd m^−2^	EL_max_ ^f^ (nm)	CIE ^g^ (x, y)
TDBA-Py	2.5	4565	12.5/11.0	10.9/6.65	11.2/10.0	471	(0.12, 0.15)
TDBA-dPy	2.9	4340	12.3/10.2	9.34/5.80	11.2/9.37	471	(0.12, 0.15)

^a^ Turn-on voltage at 1 cd m^−2^. ^b^ Maximum luminance. ^c^ Current efficiency. ^d^ Power efficiency. ^e^ External quantum efficiency. ^f^ Maximum electroluminescence wavelength. ^g^ Commission Internationale de l’Eclairage.

## Data Availability

The original contributions presented in this study are included in the article/Supplementary Materials.

## Supplementary Materials

The following supporting information can be downloaded at https://www.mdpi.com/article/10.3390/molecules31203768/s1, Figure S1: ^1^H NMR of 4,4′-((2,5-dibromo-1,3-phenylene)bis(oxy))bis(tert-butylbenzene); Figure S2: ^1^H NMR of 7-bromo-2,12-di-tert-butyl-5,9-dioxa-13b-boranaphtho[3,2,1-de]anthracene; Figure S3: ^1^H NMR of 2,12-di-tert-butyl-7-(4,4,5,5-tetramethyl-1,3,2-dioxaborolan-2-yl)-5,9-dioxa-13b-boranaphtho[3,2,1-de]anthracene; Figure S4: ^1^H NMR of 3-(3-bromophenyl)pyridine; Figure S5: ^1^H NMR of 3-(3-(4,4,5,5-tetramethyl-1,3,2-dioxaborolan-2-yl)phenyl)pyridine; Figure S6: ^1^H NMR of TDBA-Py; Figure S7: ^13^C NMR of TDBA-Py; Figure S8: ^1^H NMR of 3,3′-(5-bromo-1,3-phenylene)dipyridine; Figure S9: ^1^H NMR of TDBA-dPy; Figure S10: ^13^C NMR of TDBA-dPy; Figure S11: HR-mass spectroscopy of TDBA-Py; Figure S12: HR-mass spectroscopy of TDBA-dPy; Figure S13: Room-temperature (filled symbols) and 77 K delayed (open symbols) photoluminescence spectra of TDBA-Py (λ_ex_ = 393 nm) and TDBA-dPy (λ_ex_ = 395 nm) in the film state; Figure S14: Normalized UV-Vis absorption spectrum of ν-DABNA (in toluene, 10^−5^ M) overlaid with the normalized PL emission spectra of TDBA-Py and TDBA-dPy as neat films (50 nm); Figure S15: Electron and hole mobilities of TDBA-Py and TDBA-dPy as a function of the square root of the electric field, obtained from the electron-only devices (EODs) and hole-only devices (HODs).
