# Supplementary material for: Meta-Pyridyl Substitution for New Blue Host Materials with Enhanced Thermal Stability and Electron-Injection Properties
Source: Molecules. 2026 Oct 20;31(20):3768. doi: 10.3390/molecules31203768 (PMC13707410; doi:10.3390/molecules31203768)
Supplement: Supplementary file 1 [file molecules-31-03768-s001.zip › molecules-4623298-supplementary.pdf]

Supplementary Information

Meta-Pyridyl Substitution for New Blue Host Materials with Enhanced Thermal Stability and Electron-Injection Properties

Jeongmin Han[1], Sangwook Park[1], Saeyoung Oh[1], Hyeonho Park[1], Kiho Lee[1], Hayoon Lee[1], Jongwook Park[1,*]

[1]*Integrated Engineering, Department of Chemical Engineering, Kyung Hee University, Gyeonggi, 17104, Republic of Korea*

[*]*Correspondence: jongpark@khu.ac.kr (J. Park)*

Contents

1. General information

2. NMR and HR-Mass

Figure S1. ^{1}H NMR of **4,4′-((2,5-dibromo-1,3-phenylene)bis(oxy))bis(tert-butylbenzene)**

Figure S2. ^{1}H NMR of **7-bromo-2,12-di-tert-butyl-5,9-dioxa-13b-boranaphtho[3,2,1-de]anthracene**

Figure S3. ^{1}H NMR of **2,12-di-tert-butyl-7-(4,4,5,5-tetramethyl-1,3,2-dioxaborolan-2-yl)-5,9-dioxa-13b-boranaphtho[3,2,1-de]anthracene**

Figure S4. ^{1}H NMR of **3-(3-bromophenyl)pyridine**

Figure S5. ^{1}H NMR of **3-(3-(4,4,5,5-tetramethyl-1,3,2-dioxaborolan-2-yl)phenyl)pyridine**

Figure S6. ^{1}H NMR of **TDBA-Py**

Figure S7. ^{13}C NMR of **TDBA-Py**

Figure S8. ^{1}H NMR of **3,3′-(5-bromo-1,3-phenylene)dipyridine**

Figure S9. ^{1}H NMR of **TDBA-dPy**

Figure S10. ^{13}C NMR of **TDBA-dPy**

Figure S11. HR-mass spectroscopy of **TDBA-Py**

Figure S12. HR-mass spectroscopy of **TDBA-dPy**

3. Supplementary figures

Figure S13. Room-temperature (filled symbols) and 77 K delayed (open symbols) photoluminescence spectra of TDBA-Py (λ_{ex} = 393 nm) and TDBA-dPy (λ_{ex} = 395 nm) in the film state.

Figure S14. Normalized UV-Vis absorption spectrum of ν-DABNA (in toluene, 10^{-5} M) overlaid with the normalized PL emission spectra of TDBA-Py and TDBA-dPy as neat films (50 nm).

Figure S15. Electron and hole mobilities of TDBA-Py and TDBA-dPy as a function of the square root of the electric field, obtained from the electron-only devices (EODs) and hole-only devices (HODs).

1. General information

The reagents and solvents used in this study were purchased from Sigma-Aldrich, Tokyo Chemical Industry (TCI), or Alfa Aesar, with a minimum purity of 98%, and were used as received without further purification. Analytical thin-layer chromatography (TLC) was performed on silica gel plates (Merck 60 F254), and silica gel (Merck 60, 230–400 mesh) was used for column chromatography.

The ^{1}H- and ^{13}C-NMR spectra were recorded on a Bruker Avance 400 spectrometer in $CDCl_3$, CD_2Cl_2, or DMSO-d_6. High-resolution mass spectra (HRMS) were recorded by electron ionization (EI+) using a GC-HRMS system consisting of a JMS-700 (JEOL, Japan) mass spectrometer coupled with a 6890 Series (Agilent) gas chromatograph. Elemental analysis (EA) was carried out using a Flash EA1112 (CE Instruments) elemental analyzer.

All quantum chemical calculations were performed with the ORCA program package using the dispersion-corrected B3LYP-D3 hybrid functional and the def2-TZVPP basis set. The ground-state (S_0) geometries were optimized by density functional theory (DFT), and the frontier molecular orbital distributions and energy levels were obtained from the optimized structures. The S_1 and T_1 energies were taken as the differences in total energy between the optimized S_1 and T_1 structures and the optimized S_0 structure, and ΔE_{ST} was obtained as $E(S_1) - E(T_1)$.

Solution samples were prepared in toluene (concentration: 1×10^{-5} M), and neat films with a thickness of 50 nm were prepared on quartz substrates by vacuum thermal evaporation at a deposition rate of 1 Å s^{-1}. UV-vis absorption spectra were measured on a Lambda 1050 UV/Vis/NIR spectrometer (PerkinElmer), and PL spectra were recorded on an LS55 luminescence spectrometer (PerkinElmer) equipped with a xenon flash tube. Absolute PLQYs were determined with a Quantaurus-QY system (Hamamatsu).

The HOMO energy levels were determined by photoelectron spectroscopy in air using an AC-2 instrument (Riken Keiki, Japan). The LUMO energy levels were calculated by adding the optical band gap to the HOMO energy level.

The glass transition temperatures (T_g) and melting temperatures (T_m) of the compounds were obtained by differential scanning calorimetry (DSC) using a Discovery DSC25 (TA Instruments) under a nitrogen atmosphere at a heating rate of 10 °C min^{-1} up to 350 °C. The decomposition temperatures (T_d) were measured by thermogravimetric analysis (TGA) using a TGA4000 (PerkinElmer), in which the samples were heated to 800 °C at a rate of 10 °C min^{-1}.

The devices were fabricated by thermal evaporation with the structure ITO/NPB (40 nm)/TCTA (15 nm)/mCP (15 nm)/host: 2 wt% ν-DABNA (20 nm)/TmPyPB (40 nm)/LiF (1 nm)/Al (200 nm). The organic layers were deposited at a rate of 1 Å s^{-1} under a pressure of 10^{-6} Torr, and the LiF and Al layers were subsequently deposited without breaking the vacuum. The emitting area was 4 mm^2. The current density–voltage–luminance (J–V–L) characteristics were measured with a Keithley 2400 source meter, and the luminance and EL spectra were measured with a Minolta CS-1000A spectroradiometer.

Electron-only devices (EODs) with the structure ITO/TmPyPB (40 nm)/host (50 nm)/TmPyPB (40 nm)/LiF (1 nm)/Al (100 nm) and hole-only devices (HODs) with the structure ITO/MoO_3 (1 nm)/host (50 nm)/MoO_3 (10 nm)/Al (100 nm) were fabricated by thermal evaporation. The electron and hole mobilities of the host materials were estimated using the modified Mott–Gurney space-charge-limited current (SCLC) method according to the equation $J = (9/8)\mu\varepsilon\varepsilon_0 V^2 d^{-3}$, where ε is the relative dielectric constant of organic semiconductors, ε_0 is the vacuum permittivity, V is $V_{appl} - V_{bi}$ (where V_{appl} is the applied voltage and V_{bi} is the work function difference between the two electrodes), and d is the thickness of the active layer.

2. NMR and HR-Mass

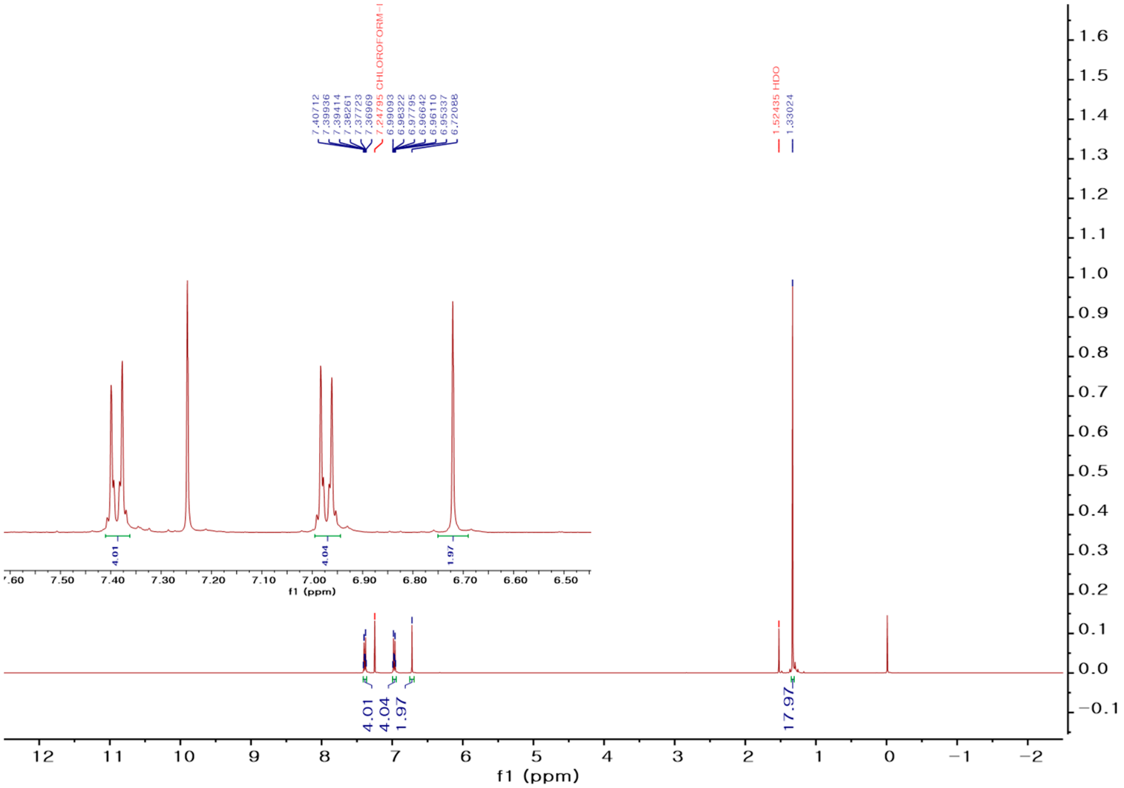

Figure S1. ^{1}H NMR of **4,4′-((2,5-dibromo-1,3-phenylene)bis(oxy))bis(tert-butylbenzene).**

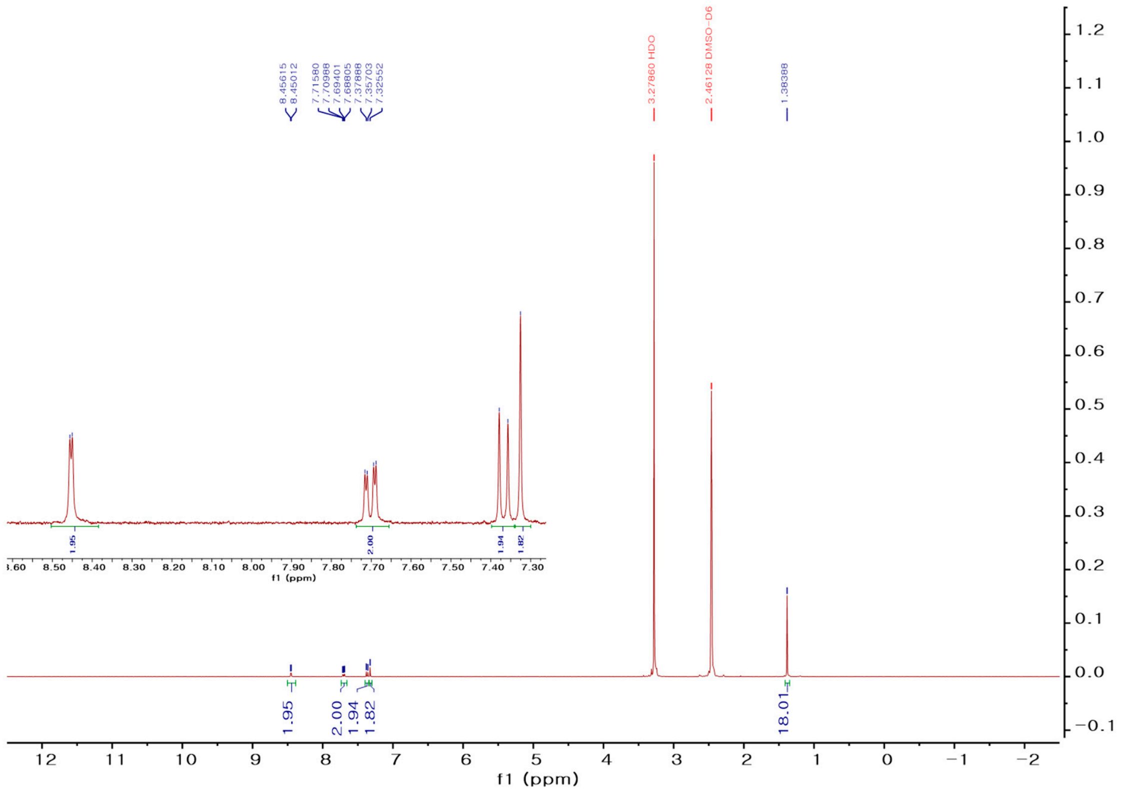

Figure S2. ^{1}H NMR of **7-bromo-2,12-di-tert-butyl-5,9-dioxa-13b-boranaphtho[3,2,1-de]anthracene.**

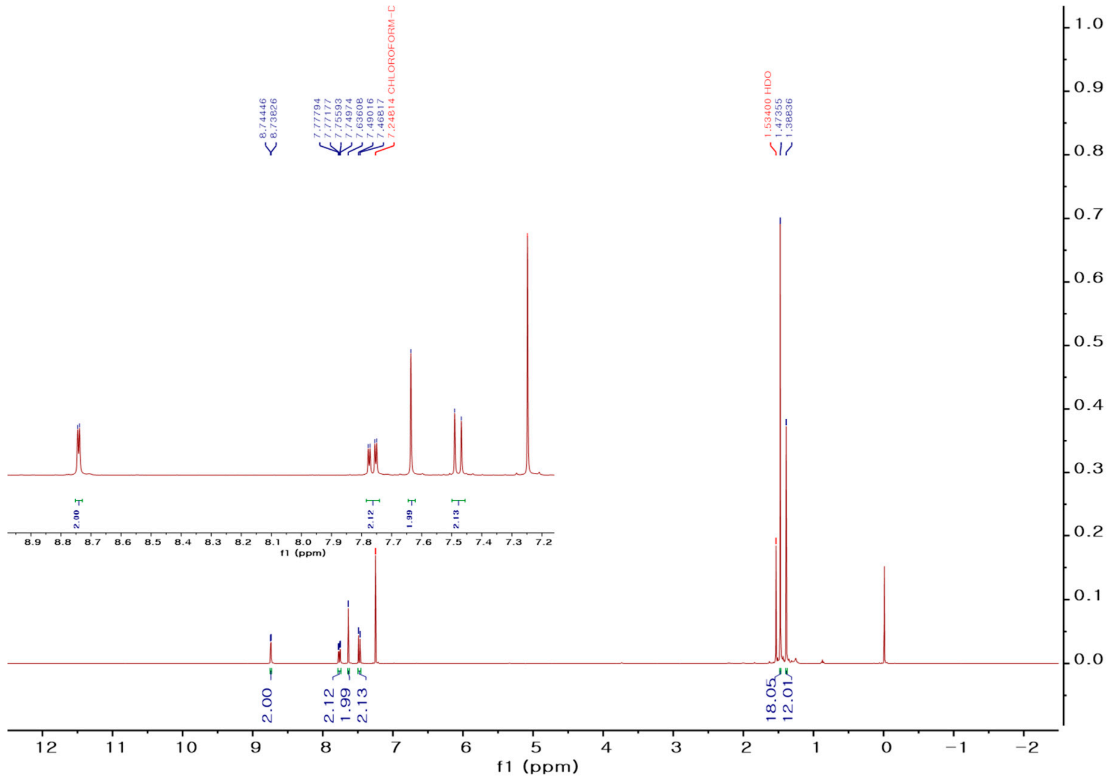

Figure S3. ¹H NMR of **2,12-di-tert-butyl-7-(4,4,5,5-tetramethyl-1,3,2-dioxaborolan-2-yl)-5,9-dioxa-13b-boranaphtho[3,2,1-de]anthracene.**

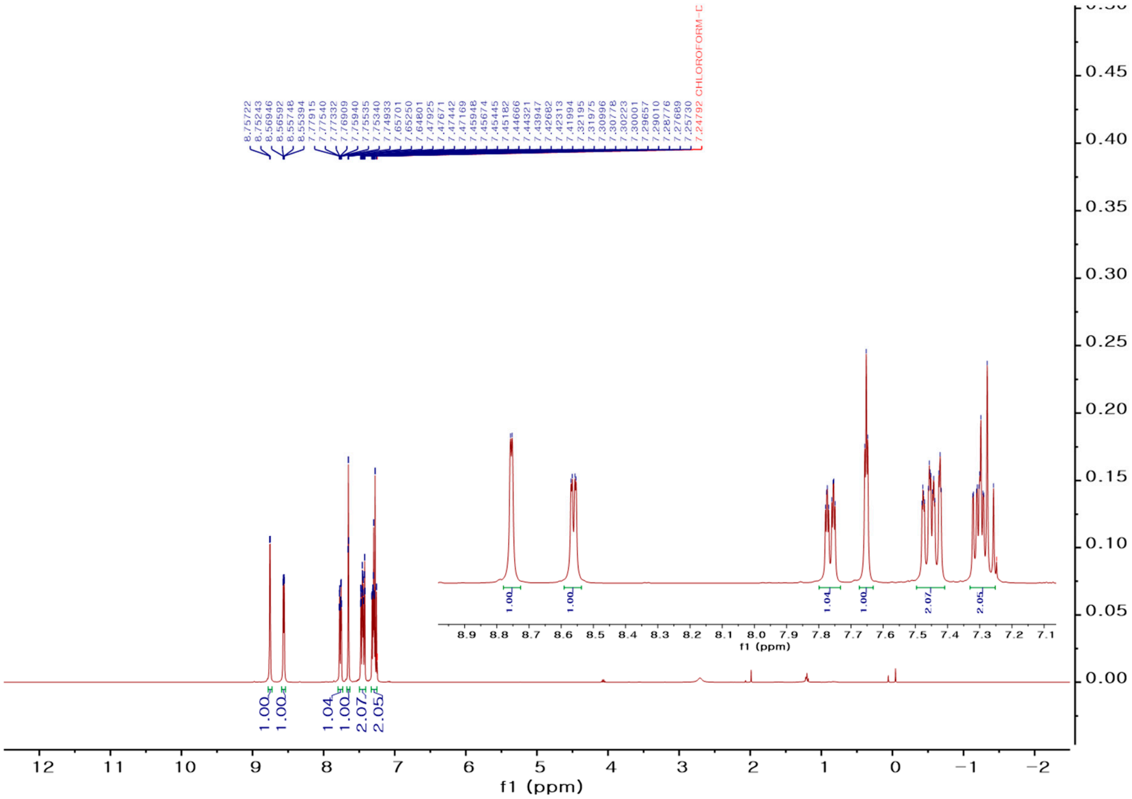

Figure S4. ^{1}H NMR of **3-(3-bromophenyl)pyridine.**

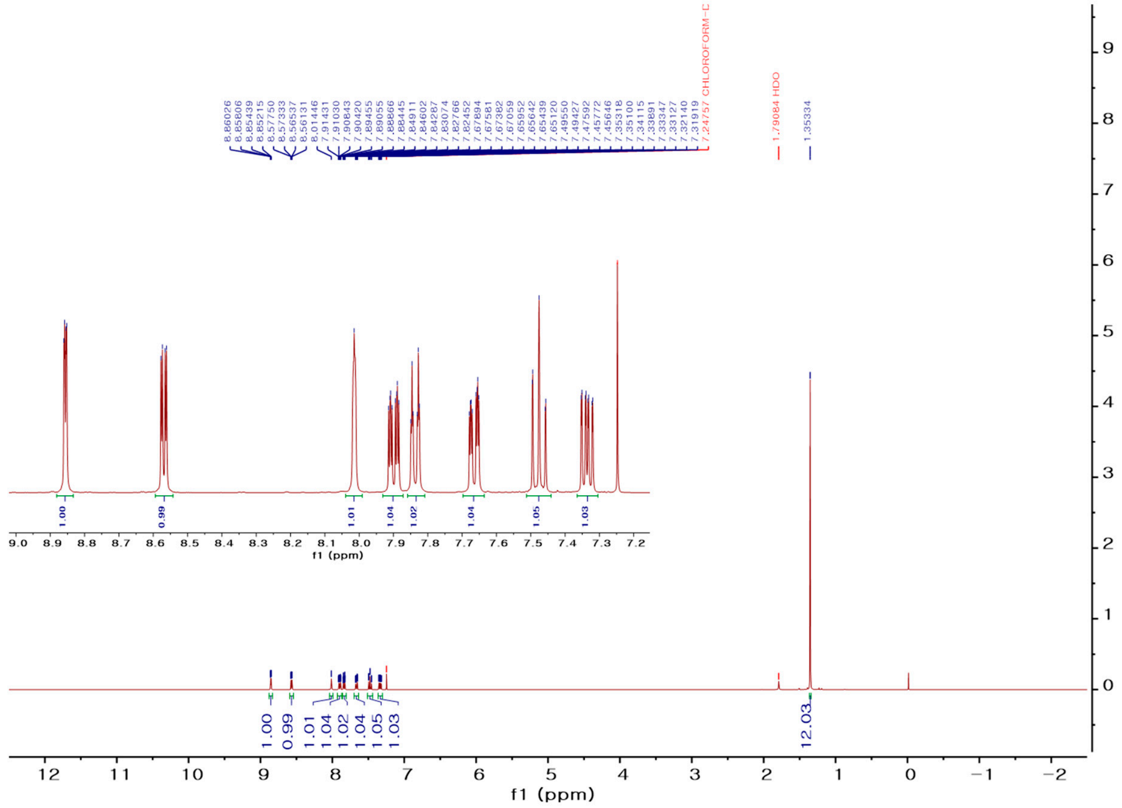

Figure S5. ^{1}H NMR of **3-(3-(4,4,5,5-tetramethyl-1,3,2-dioxaborolan-2-yl)phenyl)pyridine.**

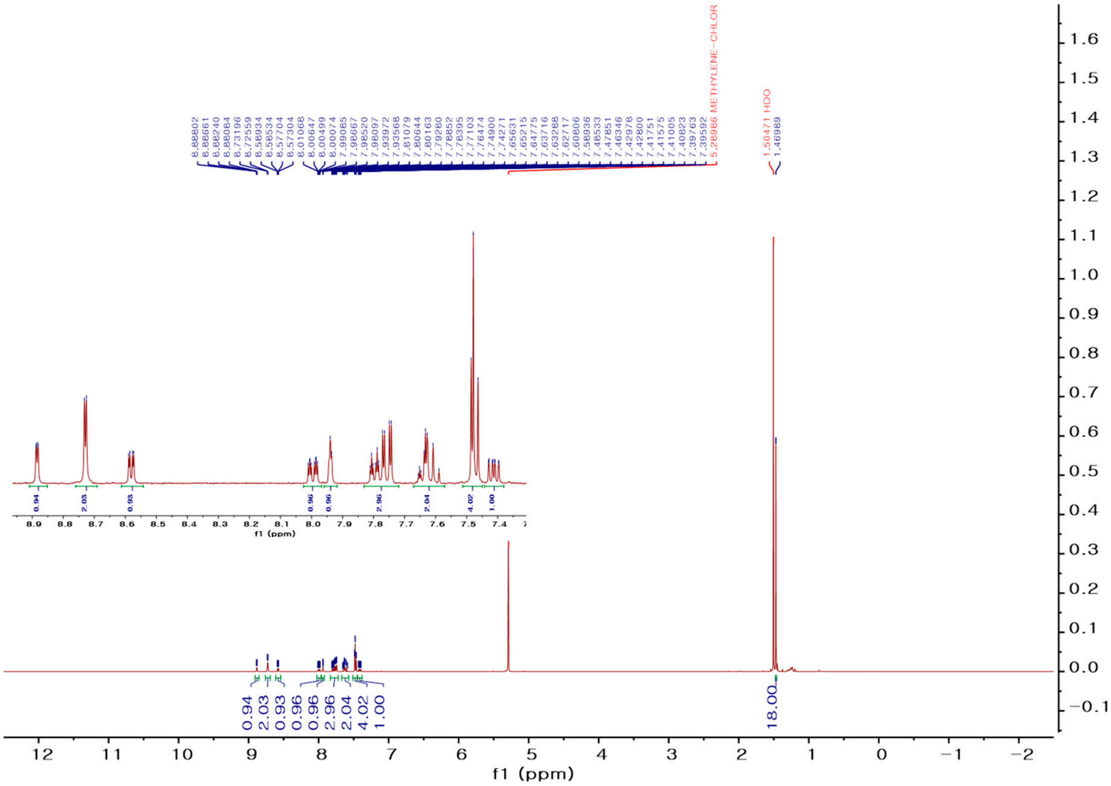

Figure S6. ^{1}H NMR of **TDBA-Py.**

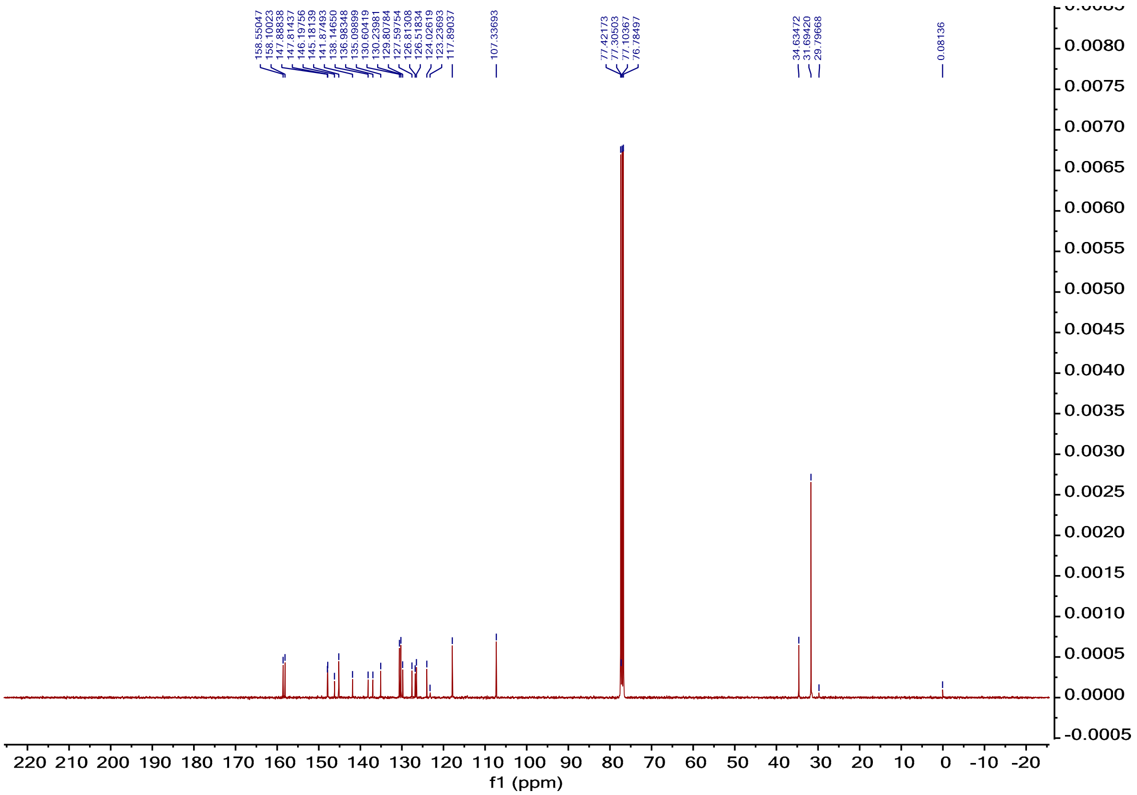

Figure S7. ^{13}C NMR of **TDBA-Py.**

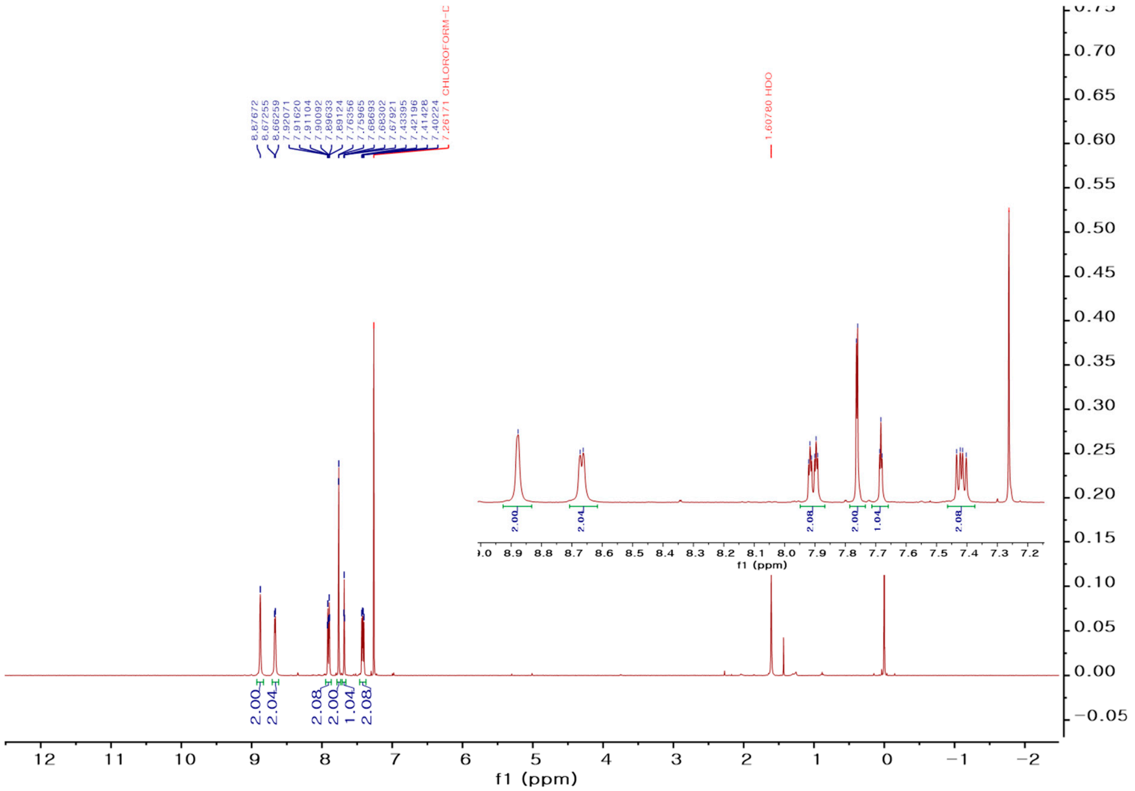

Figure S8. ^{1}H NMR of **3,3′-(5-bromo-1,3-phenylene)dipyridine.**

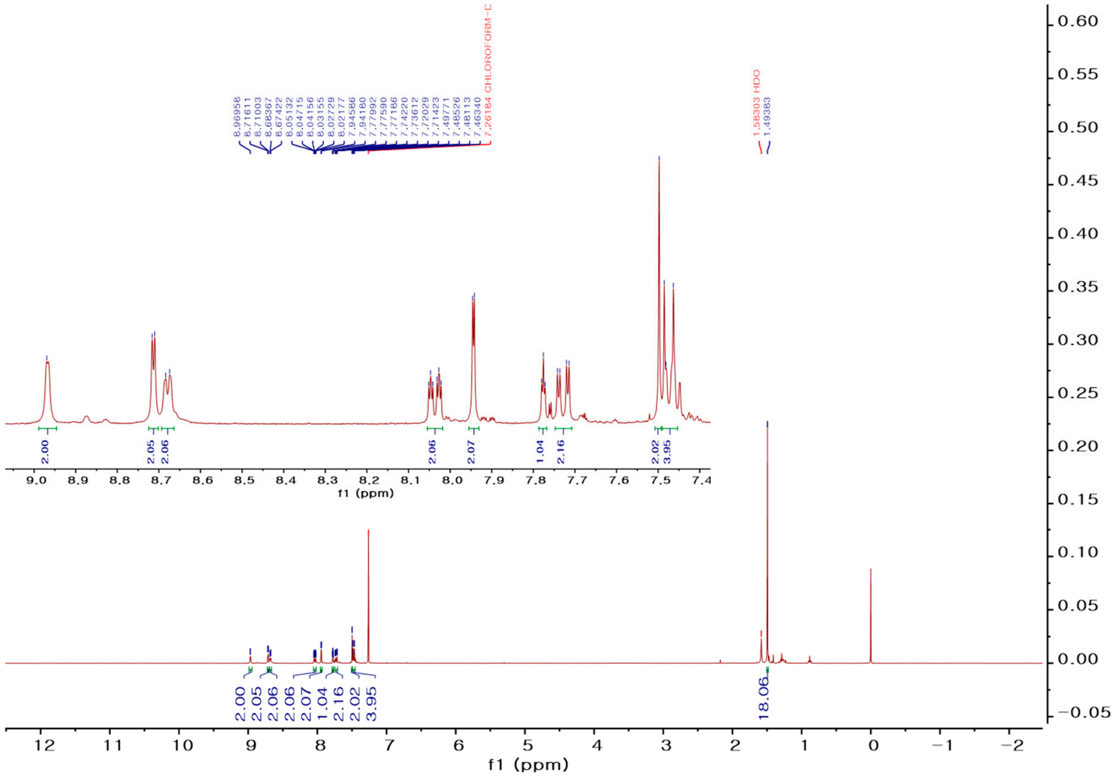

Figure S9. ^{1}H NMR of **TDBA-dPy.**

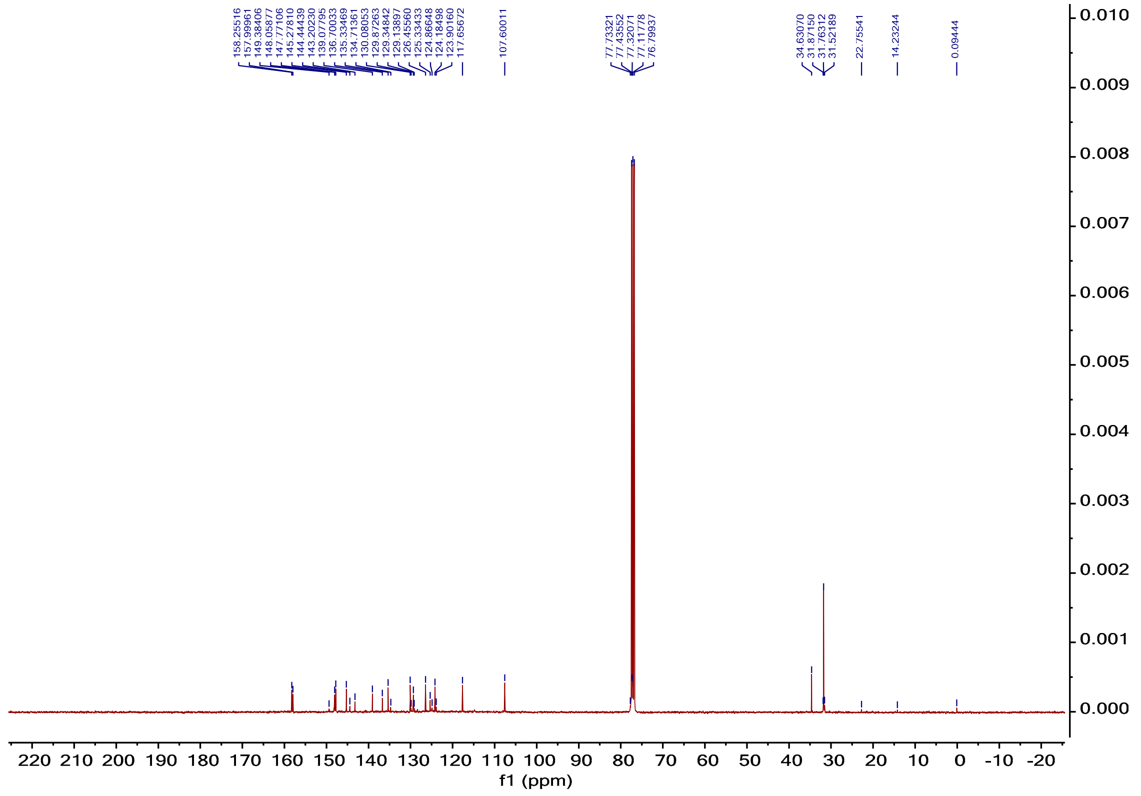

Figure S10. ^{13}C NMR of **TDBA-dPy.**

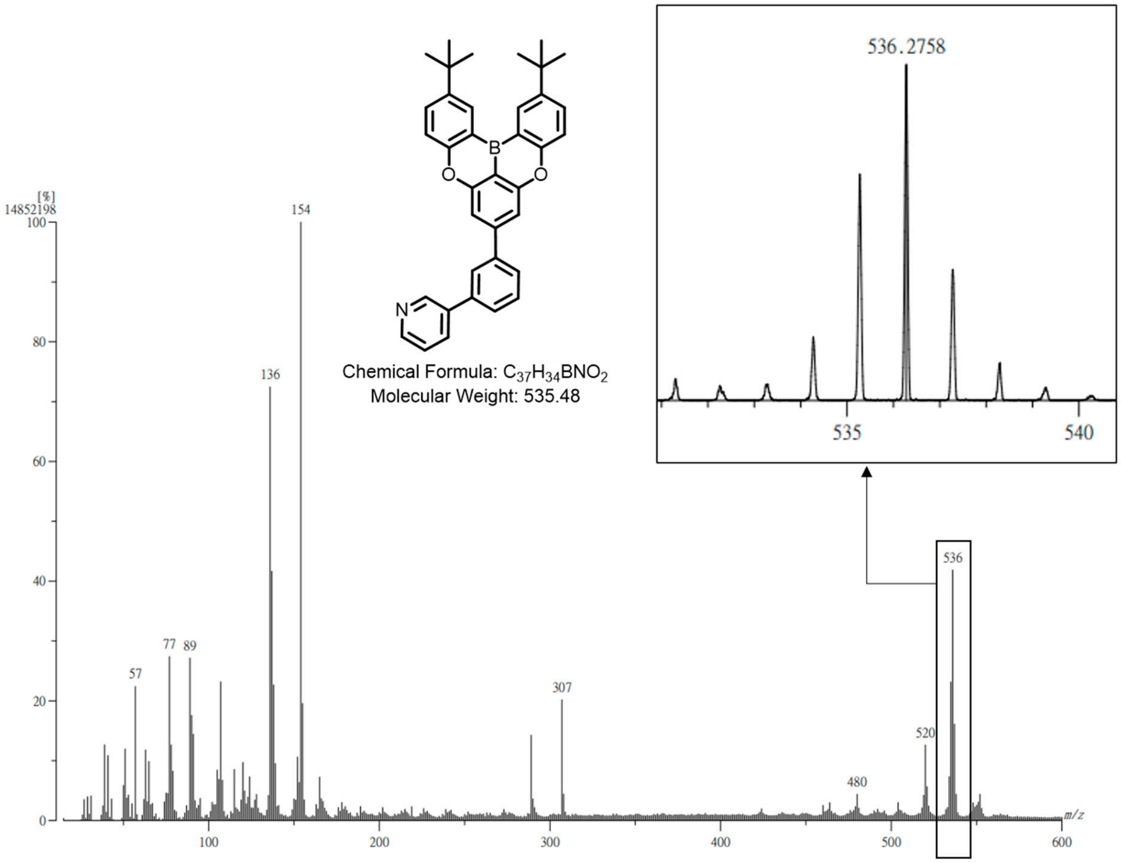

Figure S11. HR-mass spectroscopy of TDBA-Py.

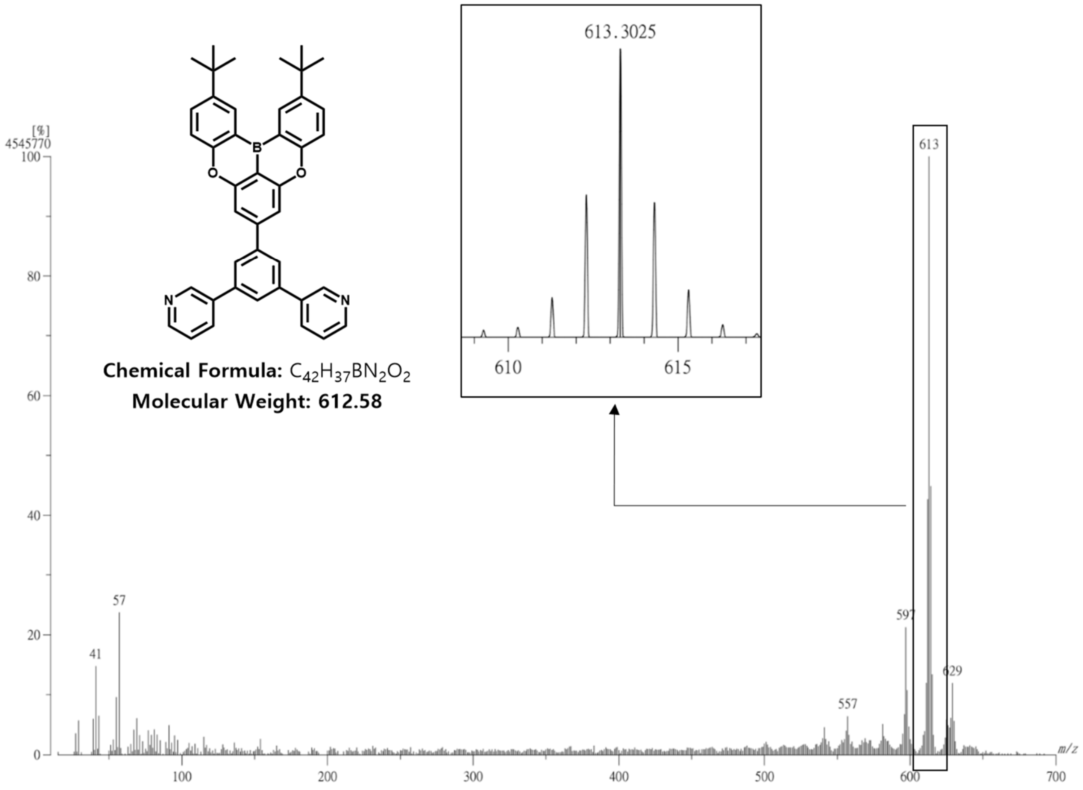

Figure S12. HR-mass spectroscopy of TDBA-dPy.

3. Supplementary figures

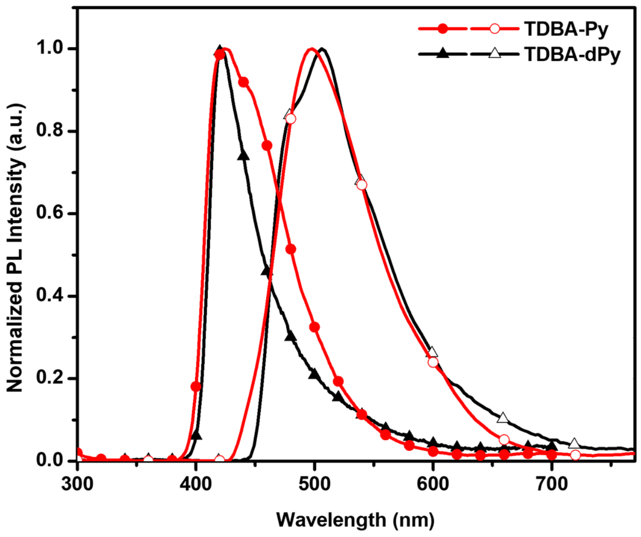

Figure S13. Room-temperature (filled symbols) and 77 K delayed (open symbols) photoluminescence spectra of TDBA-Py (λ_{ex} = 393 nm) and TDBA-dPy (λ_{ex} = 395 nm) in the film state.

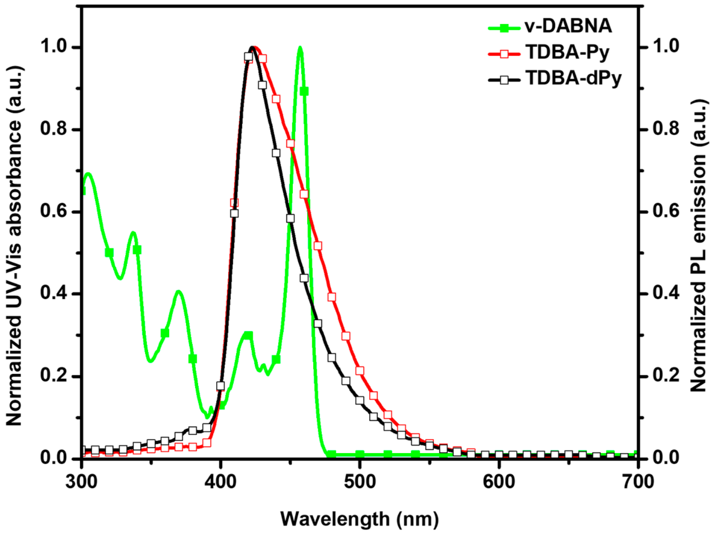

Figure S14. Normalized UV-Vis absorption spectrum of ν-DABNA (in toluene, 10^{-5} M) overlaid with the normalized PL emission spectra of TDBA-Py and TDBA-dPy as neat films (50 nm).

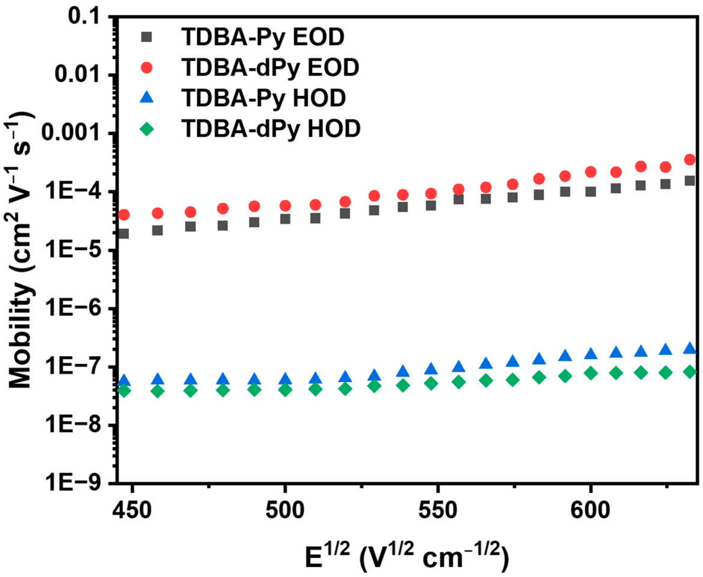

Figure S15. Electron and hole mobilities of TDBA-Py and TDBA-dPy as a function of the square root of the electric field, obtained from the electron-only devices (EODs) and hole-only devices (HODs).
